# Manual Acupuncture or Combination of Rehabilitation Therapy to Treat Poststroke Dysphagia: A Systematic Review and Meta-Analysis of Randomized Controlled Trials

**DOI:** 10.1155/2022/8803507

**Published:** 2022-10-15

**Authors:** Hailun Jiang, Qiang Zhang, Qi Zhao, Hao Chen, Xi Nan, Miao Liu, Chunsheng Yin, Wei Liu, Xiaonong Fan, Zhihong Meng, Yuzheng Du

**Affiliations:** ^1^First Teaching Hospital of Tianjin University of Traditional Chinese Medicine, Tianjin 300193, China; ^2^Tianjin University of Traditional Chinese Medicine, Tianjin 301617, China; ^3^National Clinical Research Center for Chinese Medicine Acupuncture and Moxibustion, Tianjin 300381, China; ^4^BeiJing Daxing District Hospital of Integrated Chinese and Western Medicine, Beijing 102600, China

## Abstract

*Backgroundand Objective*. Poststroke dysphagia is one of the most common stroke complications with high morbidity and long course, while acupuncture treatment is easily accepted by patients due to its reliability, feasibility, simple operation, low price, and quick effect. Our objective was to evaluate the efficacy of manual acupuncture in poststroke dysphagia patients. *Methods*. Databases including Medline, Web of Science, PubMed, Cochrane Library databases, EMBASE, CNKI (China National Knowledge Infrastructure), WanFang (WanFang Database), and VIP (Chongqing VIP) were searched from inception until Aug 19, 2022. Data were analyzed using Revman 5.3, Stata 14.0, and TSA 0.9.5.10 Beta software. Evidence quality evaluation was performed by using GRADE profiler 3.6. *Results*. A total of 33 randomized control trials (RCTs) enrolled 2680 patients. Meta-analysis results revealed that compared to rehabilitation, acupuncture decreased water swallow test (WST) and standard swallowing assessment (SSA) scores. Meanwhile, in contrast to rehabilitation alone, integration of acupuncture with rehabilitation effectively decreased WST and SSA scores; improved swallowing scores of videofluoroscopic swallowing study (VFSS), swallowing scores of Fujishima Ichiro, Barthel index (BI), and swallowing quality of life questionnaire (SWAL-QOL); reduced the aspiration rates as well as aspiration pneumonia; and shortened the duration of empty swallowing and the duration of 5 mL water swallowing. Pooled analysis did not reveal any significant differences in dysphagia outcome severity scores (DOSS) (*p*=0.15 > 0.05*p*) between the acupuncture group combined with rehabilitation group and the rehabilitation group alone. After the risk-of-bias assessment, these studies were not of low quality, except in terms of allocation concealment and blindness. Evidence quality evaluation showed that allocation concealment and blindness led to a downgrade and primary outcomes' evaluation of acupuncture combined with rehabilitation were ranked as moderate-quality evidence while acupuncture alone was ranked as low-quality. *Conclusion*. This meta-analysis provided positive pieces of evidences that acupuncture and acupuncture combined with rehabilitation were better than using rehabilitation alone in the treatment of poststroke dysphagia.

## 1. Introduction

Dysphagia, whose typical clinical manifestations include sialorrhea, bradymasesis, coughing, and choking when drinking water or eating, is prevalent among stroke patients [1]. A cohort study in South London revealed a 15%–37% prevalence rate of dysphagia among first-ever stroke patients who were recorded in the South London Stroke Register between 2001 and 2018 [2]. An Asian study reported a 36.3% incidence of dysphagia among stroke patients [3]. Potentially, dysphagia affects the quality of life, increases the occurrence of malnutrition, and prolongs hospital stay. Moreover, dysphagia patients are likely to suffer from pneumonia, resulting in death [4].

The current professional rehabilitation therapies include exercises that improve the coordination of muscle movements in the mouth and throat. Besides, applications of nasogastric tubes remain a priority for severe dysphagia patients with high aspiration risks [5]. Long periods of rehabilitation are difficult to sustain while exercises require close monitoring, leading to additional financial and manpower burdens. The placement of a nasogastric tube through the nose of a patient is associated with pain and bad postoperative memories. Additionally, a limited number of drugs, including capsaicin, nifedipine, and methylprednisolone have been reported to treat dysphagia. However, their therapeutic actions and long-term effects remain unclear [6–8]. Thus, alternative therapies, including acupuncture, neuromuscular electrical stimulation (NMES), transcranial magnetic stimulation (TMS), and balloon dilation among others, are easily accepted by patients [9]. However, these new rehabilitation technologies will cause more or less pain and discomfort to patients. Instruments such as NMES and TMS require the hospital to purchase corresponding instruments, and the treatment costs are high, making it difficult for primary hospitals' application. In addition, due to the lack of clinical research on NMES and TMS, parameters such as stimulation target selection, electrical stimulation frequency, duration, and course of treatment are still unclear [10, 11]. Pain and other discomforts will occur during the application of balloon dilatation and the course of treatment is generally more than 15 days, which will cause psychological pressure on patients. Acupuncture was first promoted by the World Health Organization (WHO) in 1979 [12] and has been extensively been used to treat various neurological diseases in China. By overcoming the serious side effects associated with chemical drugs, acupuncture is considered a “natural, green, and time-honored” therapy that is accepted by a majority of patients because of its reliability, feasibility, simple operation, low price, and instant effects [13].

However, the existing systematic reviews in some aspects should be improved. For instance, the included studies are of low quality, the level of clinical evidence cannot be established, and acupuncture methods, as well as acupoints in different studies, significantly vary. A recent review on swallowing therapy [14] from the Cochrane database put forward that acupuncture could not improve the swallowing capacity of patients. However, there was significant heterogeneity in the included articles and no corresponding explanation was given, moreover, the review [14] mentioned that the quality of evidence ranged from “very low” and “low”. The topic is of importance to clinicians and policymakers because the significance of unconventional treatments, such as acupuncture is controversial. Therefore, we aimed at providing higher-quality evidence and at exploring the clinical efficacy of acupuncture on poststroke dysphagia. We only included high-quality RCTs (the modified Jadad scores were equal to or above 4 points). Further, “manual” acupuncture and locations of acupoints were specified to minimize clinical heterogeneities.

## 2. Materials and Methods

### 2.1. Study Registration

We conducted a protocol of systematic review and meta-analysis following preferred reporting items for systematic reviews and meta-analyses protocol (PRISMA-P). Meanwhile, the study was registered on the PROSPERO (International prospective register of systematic reviews) on July 8, 2021, and the registration number is CRD42021258346.

### 2.2. Search Strategy

Two independent reviewers (Jiang. HL and Zhang. Q) searched databases including Medline, Web of Science, PubMed, Cochrane Library databases, EMBASE, CNKI (China National Knowledge Infrastructure), WanFang (WanFang Database), and VIP (Chongqing VIP) from inception until Aug 19, 2022, and found no language restriction. Based on the characteristics of each database to develop the corresponding retrieval strategy, the following English keywords were used|: (stroke *∗* OR Poststroke OR Cerebrovascular OR CVA *∗* OR Apoplexy OR Vascular Accident *∗* OR brain OR Cerebral *∗*) and (Point *∗* OR Acupuncture OR Acupoint *∗*) and (Swallowing Disorder *∗* OR Dysphagia OR Deglutition Disorder *∗*) and (Randomized OR RCT OR Randomly) and (Trial *∗*). The search strategy is listed in Table S1.

### 2.3. Inclusion Criteria

The inclusion criteria for the selected literature were as follows: (i) patients with dysphagia after stroke; (ii) clinical randomized controlled trials comparing manual acupuncture with rehabilitation therapy for the treatment of poststroke dysphagia; the manual acupuncture study group included acupuncture alone or acupuncture coupled with rehabilitation therapy to treat dysphagia; the control group was treated with rehabilitation therapy; (iii) in duplicated published articles, the one with more complete data was included; and (iv) literature that included acupoints located around the nape, neck, or throat.

Note: the diagnostic criteria for poststroke dysphagia refers to “Diagnostic Criteria of Cerebrovascular Diseases in China (version 2019) [15]” and “European Stroke Organization and European Society for Swallowing Disorders Guideline for the Diagnosis and Treatment of Poststroke Dysphagia [16].” Clinical manifestations include stroke patients choking on drinking water or voice changes, dysarthria, abnormal gag reflex, and cough after eating.

Rehabilitation therapy for swallowing disorders includes indirect training and direct training [17]. Direct training is related to the eating process. Indirect training includes the following: (1) breathing training, (2) oral exercise training, (3) oral sensorimotor training, (4) vocal cord closure training (5) supraglottic swallowing and ultrasound supraglottic swallowing, (6) Mendelsohn maneuver, (7) Shaker training, (8) Masako technique, (9) K-point stimulation, (10) low-frequency electrical stimulation, (11) swallowing apraxia training, (12) esophageal dilation, and (13) intermittent oroesophageal tube feeding.

### 2.4. Exclusion Criteria

The exclusion criteria were as follows: (i) articles whose full literature was unavailable and (ii) if the quality of the article, as evaluated by the modified Jadad scale, was rated as low quality (Jadad ＜ 4), then it was excluded.

### 2.5. Outcome

#### 2.5.1. Primary Outcomes

The primary outcomes were as follows:

  Water swallow test (WST)
  Video fluoroscopic swallowing study (VFSS)

#### 2.5.2. Secondary Outcomes

The secondary outcomes were as follows:

  Standard swallowing assessment (SSA) scores
  Swallowing scores of Fujishima Ichiro
  The rates of aspiration
  The rates of aspiration pneumonia
  The dysphagia outcome severity score (DOSS)
  Barthel index (BI)
  Swallowing quality of life questionnaire (SWAL-QOL)
  Duration of empty swallowing
  Duration of 5 mL water swallowing

### 2.6. Data Extraction

Two independent reviewers (Jiang. HL and Zhang. Q) searched and screened the works of the literature and then extracted the general information of the included trials, involving the name of the first author, year of publication, source of diagnosis, sample size, age of participants, RCTs districts, types of stroke, duration of dysphagia after stroke, intervention measures, the course of the intervention, outcome indicators, and the information about acupuncture treatment (including reinforcing and reducing, acupoints, and needle retaining time). If any inconsistency is being raised up, then the decision would be made through discussion, and if the discrepancies still persisted, then the third reviewer (Zhao Q) would make the final decision.

### 2.7. Risk-of-Bias Assessments

Each study was classified as “low,” “high,” or “unclear risk of bias” at the following items: ①random sequence generation (selection bias), ②allocation concealment (selection bias), ③ blinding of participants and personnel (performance bias), ④blinding of outcome assessment (detection bias), ⑤ incomplete outcome data (attrition bias), ⑥ selective reporting (reporting bias), and ⑦ Other bias. Two independent reviewers (Jiang. HL and Zhang. Q) evaluated the methodological quality and the risk of bias of the included RCTs separately and discussed on resolving the disagreements, based on the Cochrane risk-of-bias criteria [18]. If two reviewers were divided over risk-of-bias assessments, then the third reviewer (Zhao Q) would be consulted to confirm the judgment and to finally reach a consensus on all items.

### 2.8. Data Synthesis and Statistical Analysis

Data synthesis was performed using the Review Manager software 5.4 (developed by the UK's International Cochrane Collaboration) and Stata 14.0 (developed by the USA's StataCorp LLC). Relative risks (RR) were used as the effect analysis statistics for dichotomous variables, while the weighted mean difference (WMD) and a 95% confidence interval (CI) were calculated for continuous variables. The chi-square test was used to establish statistical heterogeneity between data of included trials; besides, *I*^2^ or Chi-square test *pp* was used to quantitatively determine heterogeneity. When *I*^2^ < 50% or chi-square test *p* ≥ 0.1*p*, heterogeneity was considered unapparent, and the fixed-effects model was applied. However, significant heterogeneity was present when *I*^2^ ≥ 50% or chi-square test *p* < 0.1*p*, and the random-effect model was applied. Then, sensitivity or subgroup analyses were performed to determine heterogeneity sources. Egger's test was performed to test for publication bias, and *p* > 0.05*p* implied the absence of publication bias. The prespecified *pp* value threshold for one primary outcome was set at *p*=0.05*p*, and for the other, it was set at *p*=0.033*p* [19]. Secondary outcomes with *p* < 0.05*p* were considered significant.

Trial sequence analysis (TSA) was performed using TSA 0.9.5.10 Beta (developed by the Copenhagen Trial Unit's Centre for Clinical Intervention Research). TSA parameter setting: I error probability of 5% and II error probability of 20% [20].

The *X*-axis represents the sample size; the *Y*-axis represents the statistics on the *Z*-value; the symmetrical green horizontal dashed lines represent the conventional boundary value (*Z* = 1.96, *p*=0.05*p* (two-sides)); the symmetrical solid red lines represent the TSA boundary value; the vertical red line represents the required information size (RIS), and the blue curve represents the cumulative *Z*-value.

In case the blue curve did not intersect with any red line, then the sample size was considered insufficient; consequently, a series of similar trials should be performed. The current sample size was considered enough if the blue curve intersected with any of the red lines.

### 2.9. Evidence Quality Evaluation

The GRADE profiler 3.6 (developed by the European Commission Marie Curie Reintegration grant EU IGR42192 to Holger Schünemann, the Cochrane Collaboration, and the Norwegian Knowledge Centre for the Health Services) was used to evaluate the quality of evidence for the primary outcome, based on the grading of recommendations, assessment, development, and evaluation (GRADE) approach [21]. As the outcome was from RCTs, the starting level of quality of evidence was high. Then, the two independent reviewers (Jiang HL and Zhang Q) separately downgraded the level from the following five aspects: imprecision (random error), unexplained heterogeneity or inconsistency of results, indirectness of evidence, study limitations (risk of bias), and publication bias, and if disagreements persisted, the third investigator (Zhao Q) was consulted to confirm the judgment so as to finally reach a consensus on all items. Ultimately, the quality of evidence was determined into the following four levels to verify the reliability and accuracy of outcomes: the highest quality, moderate quality, low, and very low [22]. Two independent reviewers (Jiang. HL and Zhang. Q) evaluated the methodological quality and the risk of bias of the included RCTs separately and discussed on resolving the disagreements, based on the GRADE handbook.

## 3. Results

### 3.1. Study Participants and Grouping

**Figure 1:**
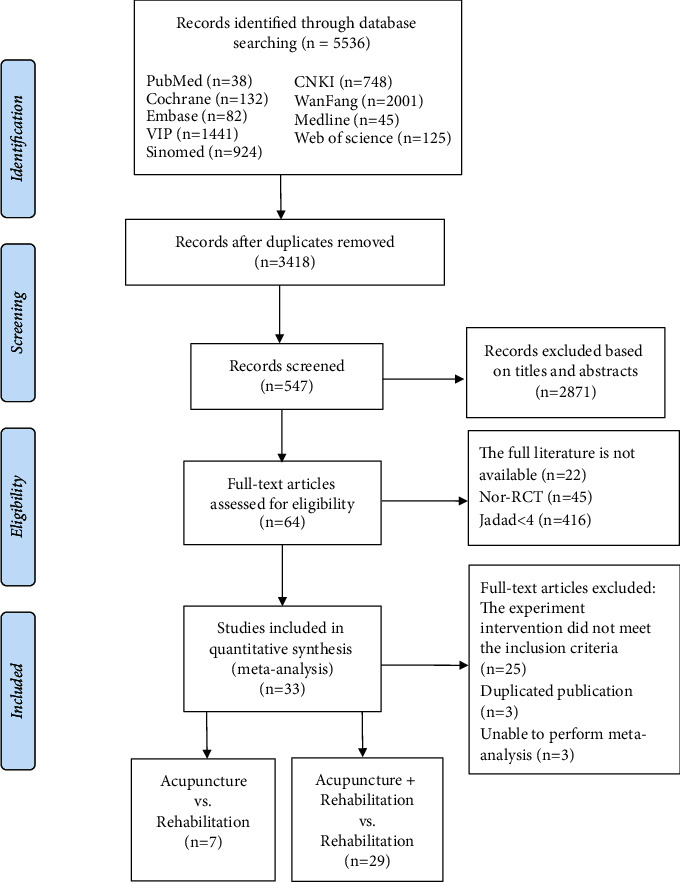
Flow chart of study identification and selection.

This study included 33 trials [23–55] involving 2,680 participants. A total of 220 participants were included in the acupuncture group, 1,289 patients were in the rehabilitation group, while 1,171 patients were in the acupuncture combined with the rehabilitation group. Besides, four RCTs [43–46] designed two groups, comprising both the acupuncture and rehabilitation groups; 26 [23–25, 28–37, 39–42, 47–55] RCTs designed two groups, comprising acupuncture + rehabilitation group and rehabilitation group; and three RCTs [26, 27, 38] designed three groups, comprising acupuncture group, rehabilitation group, and acupuncture + rehabilitation group. The process is shown in Figure 1.

### 3.2. Risk-of-Bias Assessments

The modified Jadad score for all these studies was ≥4.

  ① Random sequence generation (selection bias): All the trials reported specific randomization methods, except Xie's study [43], thus one was an “unclear risk” of selection bias, and the others were regarded as having a “low risk” of selection bias.
  ② Allocation concealment (selection bias): A total of nine [26, 27, 30, 31, 33, 42, 49, 52, 54] trials provided the methods of allocation concealment, therefore, these trials were considered to have a “low risk” of selection bias. The remaining 24 trials did not indicate the allocation concealment and were regarded as having an “unclear risk”.
  ③ Blinding of participants and personnel (performance bias) and blinding of outcome assessment (detection bias): Five [36, 48, 49, 52, 54] trials reported blindness; the outcome assessor in these trials were blinded and were considered to have a low risk of performance bias. Meanwhile, Xie's study [43] indicated blindness without specific measures, therefore, the trial was considered to have an “unclear risk” of performance bias. Given that blindness may have a certain impact on the outcome assessment, 28 trials did not indicate blindness and were considered to have an “unclear risk” performance of bias since most of the indicators were easily unaffected by psychological suggestions.
  ④ Incomplete outcome data (attrition bias): All of the studies provided the causes and numbers of lost patients at follow-up. A total of 100 patients in 16 trials were excluded after they were lost to follow-up and ITT analysis was not used; they were considered to have an “unclear risk” attrition of bias since the lost follow-up rate was less than 15%.
  ⑤ Selective reporting (reporting bias): Only one trial [36] conducted clinical registration and it was difficult to evaluate the reporting bias. Thus, we assumed that the reporting bias was at a low risk only after an ethical review board had reviewed the report. Therefore, in selective reporting, 14 trials had a low risk of reporting bias.
  ⑥Other bias: Any other bias source was not detected, therefore, all the trials were considered to have a “low risk” of bias.

**Figure 2:**
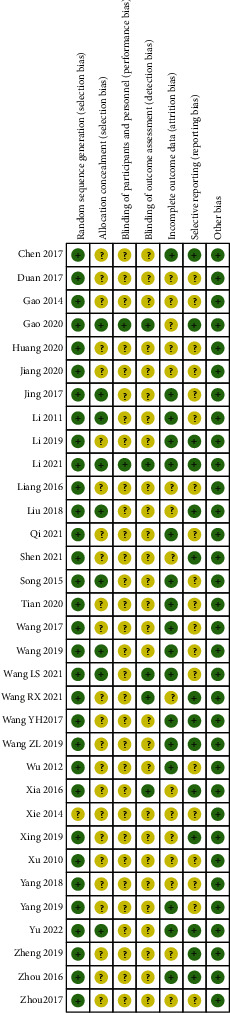
Risk of bias assessment for included studies.

Overall, the quality of these trials was not low, especially in terms of allocation concealment, and blindness is low. The blindness of manual acupuncture is a prevalent problem in clinical acupuncture trials, therefore, additional mechanisms are necessary to overcome it. The bias risk assessment is presented in Figure 2.

### 3.3. The Basic Characteristics of the Inclusion Study

#### 3.3.1. Characteristics of PICO Summarized in Table 1

**Figure 3:**
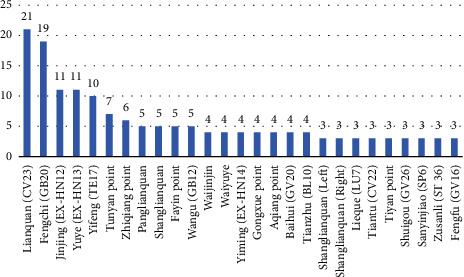
Acupoint frequency map.

Characteristics of manual acupuncture are summarized in Table 2 and Figure 3. A total of 72 acupoints were involved, and Figure 3 shows acupoints that were used greater than or equal to 3 times. Lianquan (CV23), Fengchi (GB20), Jinjing (EX-HN12), Yuye (EX-HN13), and Yifeng (TE 17), which are mainly distributed in the superior border of hyoid bone, tongue and neck, are frequently selected for stimulation. In 33 RCTs, their acupoint frequencies were 21, 19, 11, 11, and 10.

#### 3.3.2. Characteristics of the Rehabilitation Training Summarized in Table 3

### 3.4. Meta-Analysis Results

#### 3.4.1. Acupuncture vs. Rehabilitation (Figures 4 and 5)

**Figure 4:**
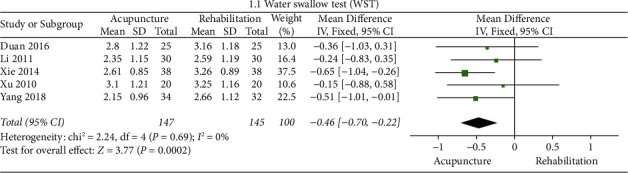
Forest plot of WST (acupuncture vs. rehabilitation).

**Figure 5:**
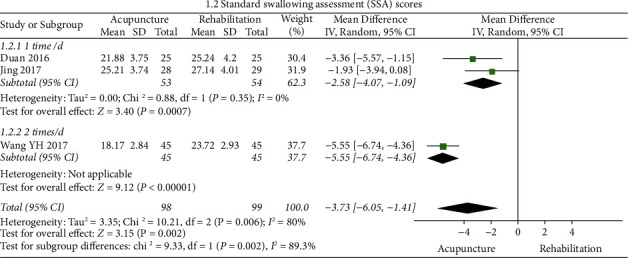
Forest plot of SSA (acupuncture vs. rehabilitation).

Compared with rehabilitation, this study found two indicators of acupuncture. Pooled results revealed significant differences in swallowing scores of WST (*p* < 0.05*p*) and SSA (*p* < 0.05() as shown in Figures 4 and 5.

#### 3.4.2. The Swallowing Scores of WST (Figure 4)

The results of the meta-analysis showed that the swallowing scores of WST of the acupuncture + rehabilitation group were lower than that of the rehabilitation group (WMD = −0.46, 95% CI (−0.70, −0.22)). In this analysis, there was no significant between-study heterogeneity (5 RCTs, *I*^2^ = 0%).

#### 3.4.3. SSA (Figure 5)

The results of the meta-analysis showed that the SSA score of the acupuncture group was lower than that of the rehabilitation group (WMD = −3.73, 95% CI (−6.05,-1.41)), and the heterogeneity of the SSA score was high (3 RCTs, *I*^2^ = 80%). The index of one study [26] crossed the invalid line (*p* > 0.05*p*), while after excluding it, heterogeneity remained apparent (*I*^2^ = 80% ⟶ *I*^2^ = 66%); meanwhile, no significant methodological heterogeneity was noted in Jing's study [26]. Subgroup analysis wasperformed based on the frequency of treatment (once/d, twice/d), where the identified frequency of treatment was a significant outcome moderator, and heterogeneity was significant between the two subgroups (*I*^2^ = 89.3%). The subgroup with treatment of twice/week (WMD = −5.55, 95% CI (6.74, −4.36)) had better outcomes than that of once/d (WMD = −2.58, 95% CI (−4.07, −1.09)). The result of the subgroup analysis revealed a tendency for WMD of SSA to increase with increasing frequencies of acupuncture treatment. However, further research is still needed due to the small number of included studies.

### 3.5. Acupuncture + Rehabilitation vs. Rehabilitation (Figures 6–16)

**Figure 6:**
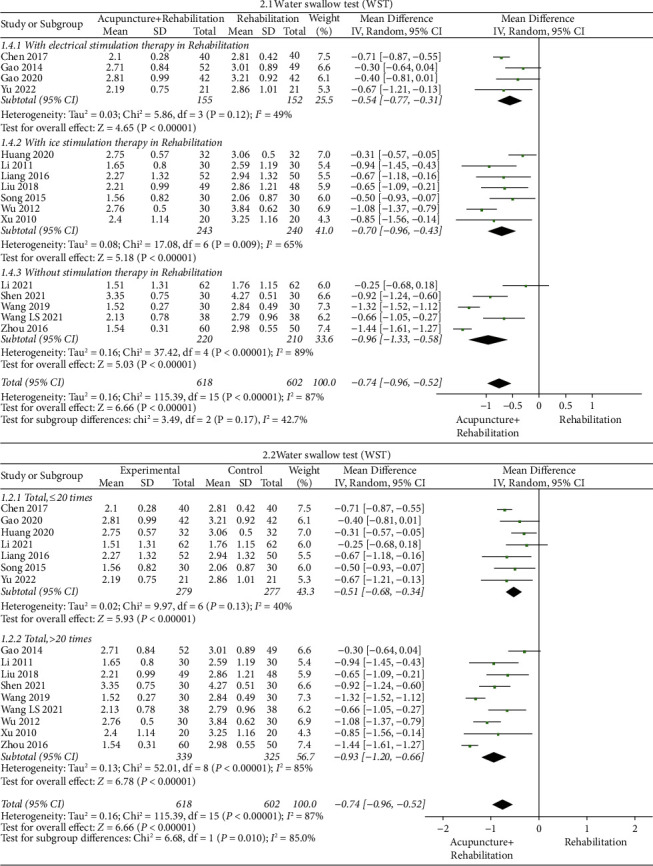
Forest plot of WST (acupuncture + rehabilitation vs. rehabilitation).

**Figure 7:**
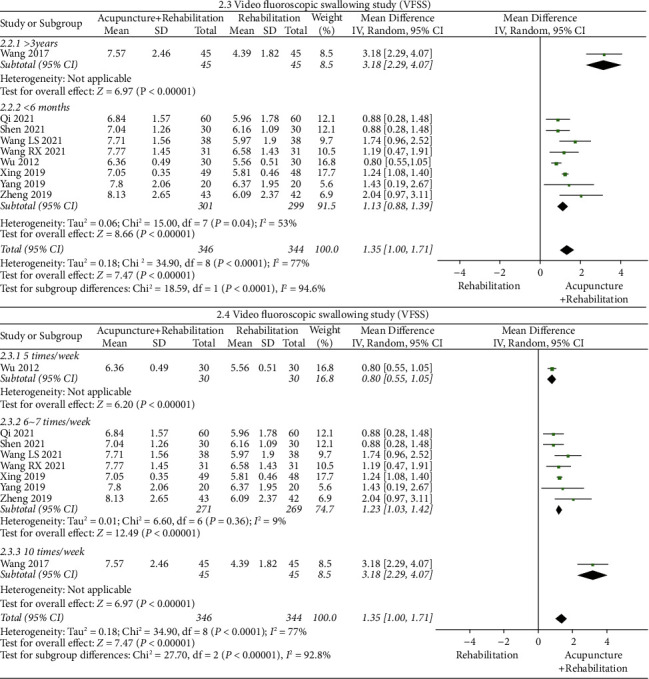
Forest plot of VFSS (acupuncture + rehabilitation vs. rehabilitation).

**Figure 8:**
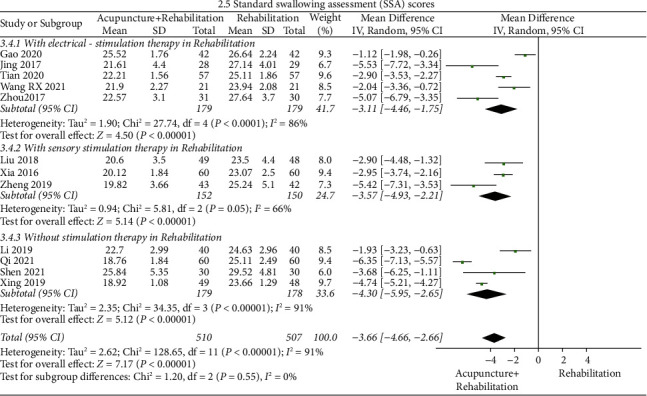
Forest plot of SSA (acupuncture + rehabilitation vs. rehabilitation).

**Figure 9:**
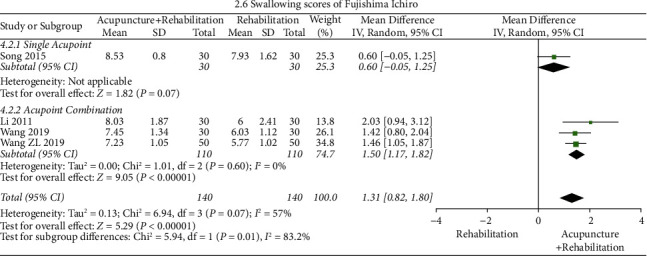
Forest plot of the swallowing scores of Fujishima Ichirowas (acupuncture + rehabilitation vs. rehabilitation).

**Figure 10:**
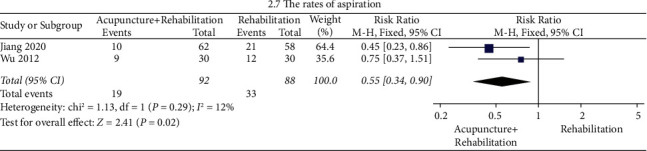
Forest plot of the rates of aspiration (acupuncture + rehabilitation vs. rehabilitation).

**Figure 11:**
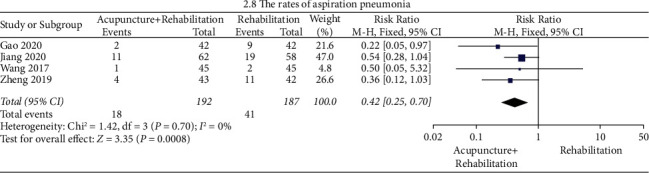
Forest plot of the rates of aspiration pneumonia. (acupuncture + rehabilitation vs. rehabilitation).

**Figure 12:**
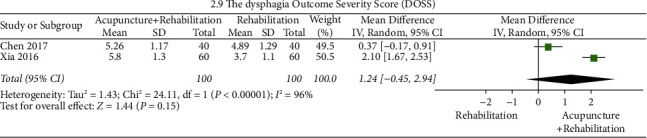
Forest plot of DOSS (acupuncture + rehabilitation vs. rehabilitation).

**Figure 13:**
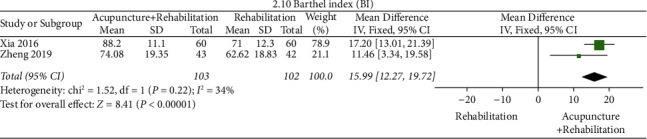
Forest plot of BI (acupuncture + rehabilitation vs. rehabilitation).

**Figure 14:**
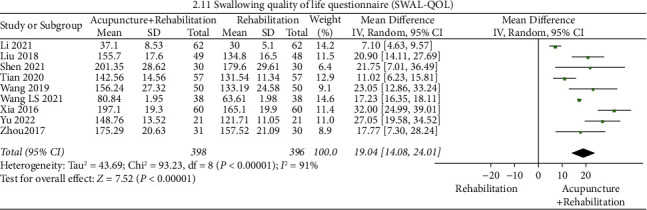
Forest plot of SWAL-QOL (acupuncture + rehabilitation vs. rehabilitation).

**Figure 15:**
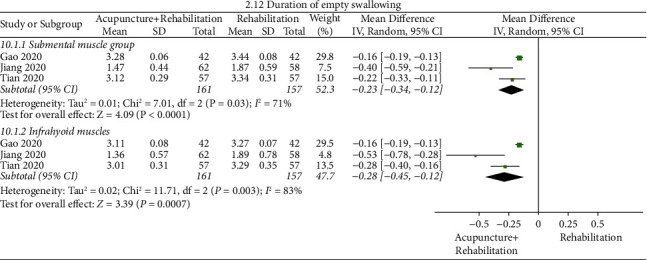
Forest plot of duration of empty swallowing (acupuncture + rehabilitation vs. rehabilitation).

**Figure 16:**
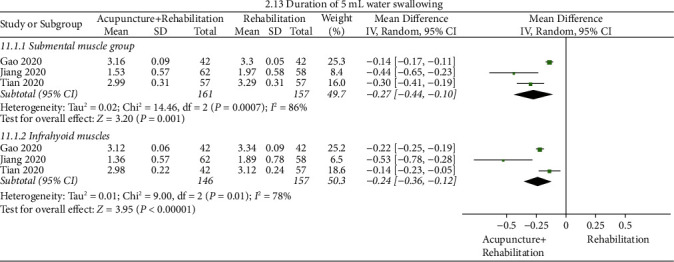
Forest plot of duration of 5 mL water swallowing (acupuncture + rehabilitation vs. rehabilitation).

Compared to rehabilitation, we found eleven indicators of the meta-analysis on acupuncture combined with rehabilitation. Pooled analysis revealed significant differences in ten indicators, including swallowing scores of WST, swallowing scores of VFSS (*p* < 0.033p), SSA, swallowing scores of Fujishima Ichiro, aspiration rates, aspiration pneumonia rates, BI, SWAL-QOL, duration of empty swallowing, and duration of 5 mL water swallowing (*p* < 0.05p) as shown in Figures 6–16.

#### 3.5.1. The Swallowing Scores of WST (Figure 6)

The results of the meta-analysis showed that the WST score of the acupuncture + rehabilitation group was lower than that of the rehabilitation group (WMD = −0.74, 95% CI (−0.96, −0.52)), and swallowing scores of heterogeneity WST were high (16 RCTs and *I*^2^ = 87%). Subgroup analysis was based on categories of stimulation therapies in rehabilitation (with electrical stimulation therapy in rehabilitation; with ice stimulation therapy in rehabilitation, and without stimulation therapy in rehabilitation), which illustrated that the mode of stimulation was a significant effect factor. The rehabilitation without stimulation therapy (WMD = −0.96, 95% CI (−1.33, −0.58)) had better outcomes than the rehabilitation with ice stimulation therapy (WMD = −0.70, 95% CI (−0.96, −0.43)) and electrical stimulation therapy (WMD = −0.54, 95% CI (−0.77, −0.31)). Subgroup analysis 2.1 indicated a tendency for WMD of WST to decrease when removing stimulation therapy in rehabilitation treatment.

Subgroup analysis 2.2 was based on the total number of treatments (≤20 times, ＞20 times). It illustrated that the total number of treatments which was greater than 20 times in subgroup (WMD = −0.93, 95% CI (−1.20, −0.66)) had better outcomes than less than 20 times' subgroup (WMD = −0.51, 95% CI (−0.68, −0.34)). Subgroup 2.2 analysis indicated a tendency for WMD of WST to decrease when the total number of acupuncture treatments was increased.

#### 3.5.2. The Swallowing Scores of VFSS (Figure 7)

The results of the meta-analysis showed that the VFSS score of the acupuncture + rehabilitation group was higher than that of the rehabilitation group (WMD = 1.35, 95% CI (1, 1.71)), and swallowing scores of VFSS's heterogeneity were high (9 RCTs and *I*^2^ = 77%). All indices were on the right of the invalid line, without significant methodological heterogeneity. Using a one-by-one exclusion method, Wang's study [32] exhibited a certain effect on *I*^2^'s variation. According to subgroup analysis 2.3 based on disease duration (>3 years,＜6 months), it illustrated that disease duration may be a significant effect factor. The subgroup with disease duration of more than 3 years (WMD = 3.18, 95% CI (2.29, 4.07)) had better outcomes than the less than 6 months' subgroup (WMD = 1.13, 95% CI (0.88, 1.39)). Subgroup analysis 2.3 suggested a tendency for WMD of VFSS to increase with a prolonged disease course.

Additionally, subgroup analysis 2.4 was performed based on the treatment frequency (5 times/week, 67 times/week, and 10 times/week), indicating that heterogeneity was derived from the treatment frequency, showing that treatment frequency may be a significant effect factor, and heterogeneity was significant among the three subgroups (*I*^2^ = 92.8% and tag 2.4). The subgroup with treatment of 10 times/week (WMD = 3.18, 95% CI (2.29, 4.07)) had better outcomes than that of 67 times/week (WMD = 1.23, 95% CI (1.03, 1.42)) and 5 times/week (WMD = 0.80, 95% CI (0.55, 1.05)). The result of the subgroup analysis revealed a tendency for WMD of VFSS to increase with increasing frequency of treatment.

The disease duration and treatment frequency all could be the sources of heterogeneity. Thus, further research studies are still needed due to the small number of included studies.

#### 3.5.3. SSA (Figure 8)

The results of the meta-analysis showed that the SSA score of the acupuncture + rehabilitation group was lower than that of the rehabilitation group (WMD = −3.66, 95% CI (−4.66, −2.66)), and the heterogeneity of SSA score was high (12 RCTs and *I*^2^ = 91%). All indices were on the left of the invalid line without significant methodological heterogeneity. Subgroup analysis was conducted based on categories of stimulation therapies in rehabilitation (with electrical stimulation therapy in rehabilitation, with sensory stimulation therapy in rehabilitation, and without stimulation therapy in rehabilitation), and it illustrated that the mode of stimulation may influence the SSA score, as the rehabilitation without stimulation therapy (WMD = −4.30, 95% CI (−5.95, −2.65)) had better outcomes than the rehabilitation with sensory stimulation therapy (WMD = −3.57, 95% CI (−4.93, −2.21)) and electrical stimulation therapy (WMD = −3.11, 95% CI (−4.46, −1.75)). However, no indication was found with regard to whether heterogeneity was derived from stimulation (tag 2.5), and there was no significant heterogeneity among the three subgroups (*I*^2^ = 0%). Subgroup analysis indicated a tendency for WMD of SSA to decrease when removing stimulation therapy during rehabilitation treatment.

#### 3.5.4. The Swallowing Scores of Fujishima Ichirowas (Figure 9)

The results of the meta-analysis showed that the swallowing scores of Fujishima Ichirowas of the acupuncture + rehabilitation group were higher than that of the rehabilitation group (WMD = 1.31, 95% CI (0.82,1.80)), and the heterogeneity was high (4 RCTs and *I*^2^ = 57%). The index of Song's study [31] crossed the invalid line (*p* > 0.05*p*), and after excluding it, the variation in heterogeneity was remarkable (*I*^2^ = 57% ⟶ *I*^2^ = 0%). Subgroup analysis was performed based on the number of acupoints (single point and acupoint combination) and it illustrated that the acupuncture prescription contained acupoint combination (WMD = 1.5, 95% CI (1.17, 1.82)) and had better outcomes than single acupoint (WMD = 0.60, 95% CI (−0.05, 1.25)).

#### 3.5.5. The Rates of Aspiration (Figure 10)

The results of the meta-analysis showed that the rates of aspiration of the acupuncture + rehabilitation group were lower than that of the rehabilitation group (RR = 0.55, 95% CI (0.34, 0.90)). In this analysis, no significant between-study heterogeneity (2 RCTs and *I*^2^ = 12%) was observed.

#### 3.5.6. The Rates of Aspiration Pneumonia (Figure 11)

The results of the meta-analysis showed that the rates of aspiration pneumonia of the acupuncture + rehabilitation group were lower than that of the rehabilitation group (RR = 0.42, 95% CI (0.25, 0.70)). In this analysis, there was no significant between-study heterogeneity (4 RCTs and *I*^2^ = 0%), and subgroup analysis was not performed.

#### 3.5.7. DOSS (Figure 12)

Pooled analysis did not reveal significant differences in DOSS (WMD = 1.31, 95% CI (0.82, 1.80), *p*=0.15 > 0.05*p*) between the groups. In this analysis, there was a significant between-study heterogeneity (2 RCTs and *I*^2^ = 96%).

#### 3.5.8. BI (Figure 13)

The results of the meta-analysis showed that the BI score of the acupuncture + rehabilitation group was lower than that of the rehabilitation group (WMD = 15.99, 95% CI (12.27, 19.72)). In this analysis, there was no significant between-study heterogeneity (2 RCTs and *I*^2^ = 34%).

#### 3.5.9. SWAL-QOL (Figure 14)

The pooled results of the meta-analysis presented that the SWAL-QOL score of the acupuncture + rehabilitation group was higher than that of the rehabilitation group (WMD = 19.04, 95% CI (14.08, 24.01)). In this analysis, there was a significant between-study heterogeneity (9 RCTs, and *I*^2^ = 91%).

#### 3.5.10. Duration of Empty Swallowing (Figure 15)

The pooled results of meta-analysis presented that the duration of empty swallowing of the acupuncture + rehabilitation group took less time than that of the rehabilitation group (WMD_1_ = −0.23, 95% CI (−0.34, −0.12), WMD_2_ = −0.28, 95% CI (−0.45–0.12)). In this analysis, a significant between-study heterogeneity (3 RCTs, *I*_1_^2^ = 71%, *I*_2_^2^ = 83%) was observed.

#### 3.5.11. Duration of 5 mL Water Swallowing (Figure 16)

The pooled results of meta-analysis presented that the duration of 5 mL water swallowing of the acupuncture + rehabilitation group took less time than that of the rehabilitation group (WMD_1_ = −0.27, 95% CI (−0.44, −0.10), WMD_2_ = −0.24, 95% CI (−0.36–0.12)). In this analysis, there was significant between study heterogeneity (3 RCTs, *I*_1_^2^ = 86%, and *I*_2_^2^ = 78%).

### 3.6. Trial Sequence Analysis (Figures 17–19)

#### 3.6.1. The TSA of Acupuncture Alone (WST)

**Figure 17:**
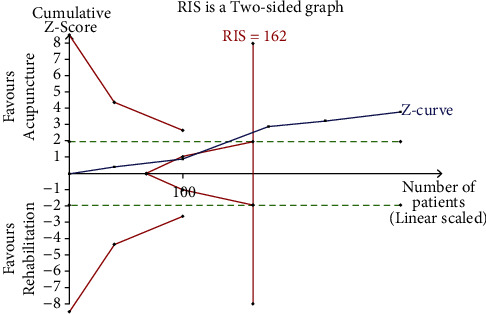
Trial sequence analysis of acupuncture alone (WST).

The TSA of acupuncture alone (WST) revealed that the cumulative Z-curve crossed the TSA boundary value when the second trial [27] was complete, met the conventional boundary value (*Z* = 1.96, *p*=0.05*p* (two-sided)) and RIS (162 cases) when the third study [43] was complete. This means that the cumulative sample size met expectations, suggesting that similar clinical trials can be terminated as shown in Figure 17.

#### 3.6.2. The TSA of Acupuncture Combined with Rehabilitation (WST)

**Figure 18:**
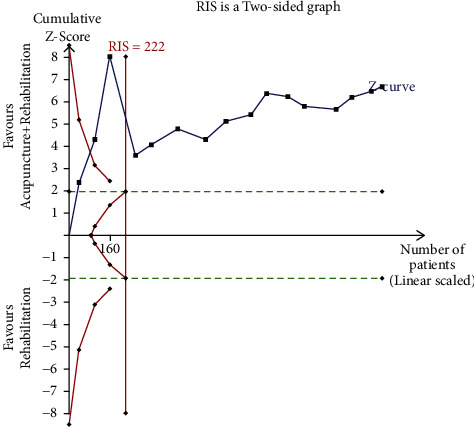
Trial sequence analysis of acupuncture + rehabilitation (WST).

The TSA of acupuncture combined with rehabilitation (WST) revealed that the cumulative Z-curve crossed the conventional boundary value (*Z* = 1.96, *p*=0.05*p* (two-sided)) when the first study [38] was complete, reached the TSA boundary value when the second study [27] was complete, and met RIS (222 cases) when the fourth study [24] was complete. This means that the cumulative sample size met the expectations, suggesting that additional similar clinical trials are unnecessary as shown in Figure 18.

#### 3.6.3. The TSA of Acupuncture Combined with Rehabilitation (VFSS)

**Figure 19:**
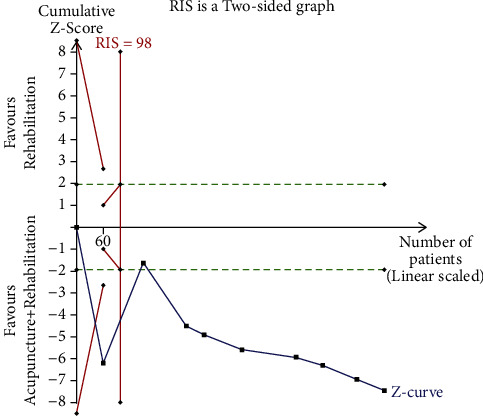
Trial sequence analysis of acupuncture + rehabilitation (VFSS).

The TSA of acupuncture combined with rehabilitation (VFSS) revealed that the cumulative *Z*-curve reached the TSA boundary value when the first study [35] was complete, met RIS (98 cases) when the second study [32] was complete, and crossed the conventional boundary value (*Z* = 1.96, *p*=0.05*p* (two-sided)) when the third study [39] was complete. This implies that the cumulative sample size met expectations, suggesting that additional similar clinical trials are unnecessary. The combination of TSA and meta-analysis reduced false-positive results further confirming the reliability of the findings. All three TSA results confirmed the benefits of acupuncture therapy in poststroke dysphagia as shown in Figure 19.

### 3.7. Safety Analysis

Thirty-three randomized controlled trials were included in this study, twelve of which reported the loss at follow-up; however, this loss at follow-up was insignificantly related to experimental research. The reasons for loss at follow-up were mostly factors, including poor patient compliance and family reasons. Furthermore, one trial [20] reported one case of cerebral infarction recurrence and one case of severe pneumonia in the treatment group. In the control group, we reported one case of cerebral infarction recurrence and two cases of severe pneumonia. Ten trials [21, 24–26, 31, 32, 48, 51–53] reported mild adverse reactions, including three cases of fainting needles, eighteen cases of subcutaneous hemorrhage, four cases of pain, 2 cases of nausea, 2 cases of inappetence, and one of them withdrew from the trial because of fainting during needles. One trial [19] reported four patients who were allergic to electrode sticks of the dysphagia treatment instrument and these patients withdrew at midway. One trial [55] reported four patients withdrew because of fainting, cannot insist on swallowing rehabilitation training and other reasons. None of the 33 trials reported any severe adverse reactions due to acupuncture and rehabilitation treatments, indicating the safety of acupuncture and rehabilitation therapies.

### 3.8. Publication Bias (Figures 20–25)

**Figure 20:**
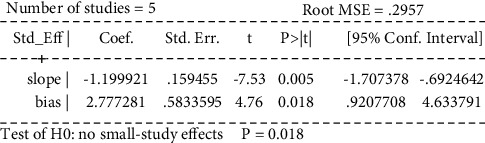
Egger's test for the WST (acupuncture vs. rehabilitation).

**Figure 21:**
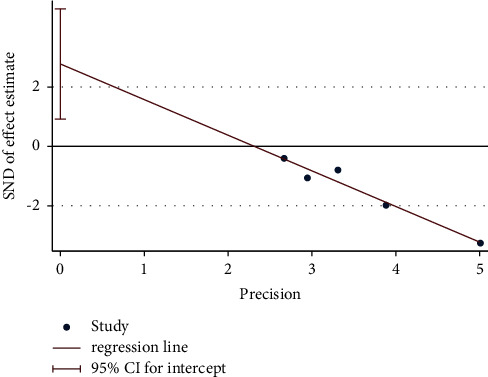
Egger graph for the WST (acupuncture vs. rehabilitation).

**Figure 22:**
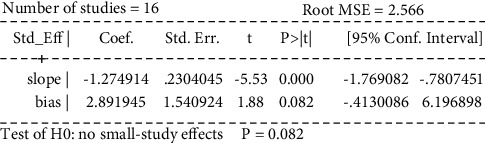
Egger's test for the WST (acupuncture + rehabilitation vs. rehabilitation).

**Figure 23:**
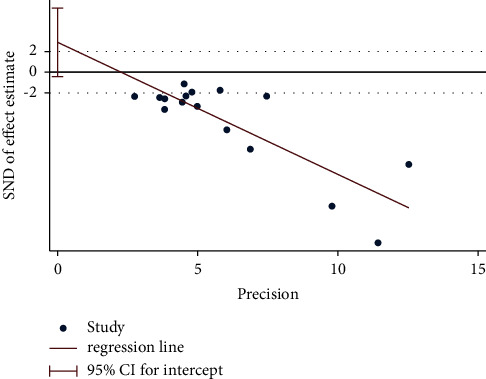
Egger graph for the WST (acupuncture + rehabilitation vs. rehabilitation).

**Figure 24:**
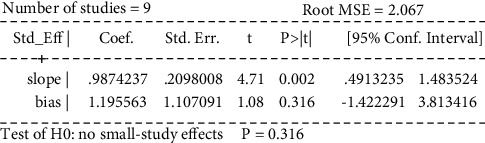
Egger's test for the VFSS (acupuncture + rehabilitation vs. rehabilitation).

**Figure 25:**
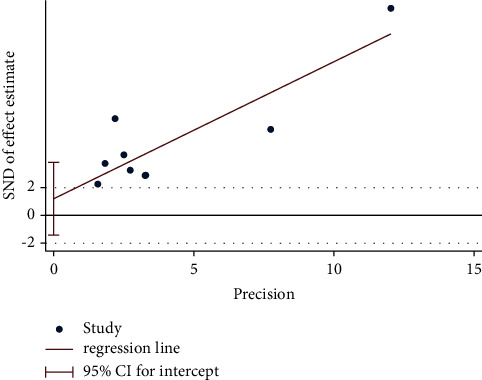
Egger graph for the VFSS (acupuncture + rehabilitation vs. rehabilitation).

Egger's test was performed to investigate the publication bias of the primary outcome. Five studies were evaluated for the WST of acupuncture alone (Egger's test: *p*=0.018*p*, Figure 20, and Egger graph, Figure 21), and the findings showed publication bias of manual acupuncture alone in treating poststroke dysphagia. Fourteen studies were evaluated for the WST of acupuncture combined with rehabilitation (Egger's test: *p*=0.082*p*, Figure 22, and Egger graph, Figure 23), and the findings showed no publication bias of manual acupuncture combined with rehabilitation therapy in treating poststroke dysphagia. Moreover, nine studies were evaluated for the VFSS of acupuncture combined with rehabilitation (Egger's test: *p*=0.316*p*, Figure 24, and Egger graph, Figure 25), and the findings showed no publication bias of manual acupuncture in treating poststroke dysphagia.

### 3.9. Sensitivity Analysis (Figures S3–S17)

A sensitivity analysis was performed to test the stability of the results. Only 1 study [36] was unstable (Figure S12). This could have been attributed to the small number of included studies containing BI or large interindividual variations in curative effects. Of course, more reasons still needs to be explored.

### 3.10. Evidence Quality Evaluation (Table 4)

The quality of evidence for primary outcomes was evaluated based on the evidence quality grading system (GRADE). Study limitations (risk of bias: most of the studies have methodological problems in allocation concealment and blindness) led to a downgrade, one outcome was ranked as low-quality evidence and two were ranked as moderate-quality evidence and the results are shown in Table 4.

## 4. Discussion

Compared to the rehabilitation group, the acupuncture group and acupuncture combined with the rehabilitation group demonstrated better effects in the treatment of poststroke dysphagia.

All included studies contained acupoints in the nape, neck, or throat areas, thereby reducing clinical heterogeneity. Besides, they were closely associated with the stimulation of neck muscles and nerves in dysphagia treatment. Acupuncture at Fengchi (GB20) point increases the amplitude of submental muscles and subhyoid muscles, indicating that acupuncture increases average muscle amplitude and muscle strength [56]; acupuncture on Lianquan (CV23) and Panglianquan stimulates the pharyngeal muscles, including the tongue muscle, hyoid muscle, pharyngeal constrictor, and superior pharyngeal constrictor; and the three acupoints are related to the hypoglossal, vagus, and glossopharyngeal nerves. Acupuncture on these three acupoints stimulates nerve motor fibers and generates nerve impulses to the cerebral cortex or the medulla oblongata swallowing center, repairs the damaged medullary arc function after stroke, and improves the swallowing function [25]. Yifeng (TE17), Wangu (GB12), and Lianquan (CV23) are associated with the vagus nerve, glossopharyngeal nerve, facial nerve, and other nerve endings. Acupuncture initiates nerve impulses and enhances nerve reflexes, repairs or rebuilds the swallowing reflex arc, and promotes swallowing functions [57, 58]. Since acupuncture is extensively used in stroke rehabilitation, multiple studies have focused on interconnections between acupuncture therapy and brain functions as well as on poststroke structural plasticity [59]. For instance, one study applied functional magnetic resonance imaging (fMRI) based on graph theory analysis. It reported that the regulatory effect of acupuncture potentially promotes the reorganization of disrupted poststroke whole-brain networks and the neural plasticity process [60]. Therefore, acupuncture regulates the peripheral nerves and the central nervous system.

In the acupuncture combined with the rehabilitation group, subgroup analyses of WST and SSA (Figure 6, tag 2.1 and Figure 8, tag 2.5) revealed that when other stimuli (including ice stimulation and electrical stimulation) were involved in rehabilitation training, and there was a tendency for the efficacy of acupuncture combined with the rehabilitation to decrease. One clinical study confirmed that there was no difference in the therapeutic effects of gustative-thermic-tactile stimulation and the addition of neuromuscular electrical stimulation [61]. Perhaps, one single stimulus is sufficient for dysphagia. Thus, we raise the question of whether more types of stimulation imply better outcomes for patients? Another RCT did not reveal any differences in therapeutic effects between neuromuscular electrostimulation therapy (NMES) and traditional dysphagia therapy (TDT such as thermal stimulation, posture adaptation, and lingual/larynx-motional exercises) and both treatments improved the symptoms of dysphagia [62]. Perhaps, electrical stimulation and TDT were able to substitute for each other. A high-quality randomized double-blind clinical trial concluded that the therapeutic effects of exercise-based swallowing therapy alone were superior to NMES [63]. Furthermore, the same article indicated the primary hypothesis that exercise-based swallowing therapy + NMES would result in superior outcomes was not upheld [63]. The abovementioned studies [61–63] opposed that more treatments mean better outcomes. In the meta-analysis, we propose that when acupuncture exists in treatment, then, stimulative treatments in rehabilitation therapies can be substituted. Multiple stimuli increase negative feelings for patients (such as pain, bradycardia, and laryngeal muscle spasms) and are an economic burden. Currently, only one study has confirmed that there are no differences in response rates of acupuncture plus neuromuscular electrical stimulation versus acupuncture alone [64]. Additional studies found that acupuncture plus electrical stimulation is better than acupuncture alone [65–69]; in contrast with the findings of this study. Due to the poor quality of the abovementioned reports [64–70], additional high-quality RCTs are necessary to explore whether stimulative treatments in rehabilitation training can be substituted by acupuncture.

In the review, there was one high-quality study [24] of acupuncture plus neuromuscular electrical stimulation versus neuromuscular electrical stimulation (Figure 6), which showed no significant differences between the two groups. Indirectly, this addresses the question above (whether more types of stimulation imply better outcomes for patients), and the answer to this is either acupuncture or electrical stimulation is sufficient. Certainly, different application parameters of NMES combined with acupuncture were out of the scope of the current study.

Subgroup analysis of VFFS revealed that acupuncture combined with rehabilitation exhibits a good curative effect on the long course of the disease and a high frequency of treatment (Figure 7, tag 2.3 and tag 2.4). With a follow-up period of 1 year, a recent clinical trial confirmed an optimum period of rehabilitation of two to three months after stroke [70]. Apart from Wang et al. [32], the other eight patients in RCTs had a disease course of two to three months. Nevertheless, due to the lack of longer follow-up and few studies with disease duration of more than 3 years, neither follow-up effect on long disease duration patients nor optimal timing of acupuncture is clear, and further research studies are needed. Subgroup analysis of VFFS indicated that without follow-up, short-term efficacy was more apparent in patients with a longer disease course. Additionally, due to the small number of included studies, it was difficult to establish whether heterogeneity was affected by the intervention or patient characteristics, or both. Besides, in the acupuncture alone group and acupuncture combined with the rehabilitation group, subgroup analyses of SSA and WST suggested the efficacy of acupuncture may be related to the acupuncture treatment dose. The dose-response relationship is a hallmark of pharmacological studies and this relationship also exists in acupuncture research. Stimulation's dose including the total number and the frequency of treatments is considered to be one of the most important components of acupuncture and it may have a great impact on the efficacy of acupuncture [71].

Swallowing scores of Fujishima Ichirowas' subgroup analysis revealed a significant heterogeneity between the two subgroups (*I*^2^ = 83.2%, Figure 5, and tag 5). Moreover, in the treatment of poststroke dysphasia, rehabilitation training combined with multiple acupoints was more effective than rehabilitation training combined with a single acupoint in the treatment of poststroke dysphasia. This is because dysphagia treatment with a single acupoint may easily induce acupuncture tolerance. Acupuncture tolerance was first proposed by professor Han et al. [72]. With advances in acupuncture research, studies have demonstrated that long-term and repeated acupuncture stimulation leads to adaptation. Besides, local receptors on acupoints are no longer sensitive [73, 74]. Therefore, avoiding long-term single acupuncture stimulation at the same acupoint is necessary and thus, acupoint combination should be considered [75].

Given that VFSS is a “gold standard” for dysphagia diagnosis and exhibits high sensitivity and specificity, and considering the economical, quick, and clear classification of the water swallow test, the water swallow test and videofluoroscopic swallowing study (VFSS) were selected as the primary outcomes of this study. Chen's study [23] used WST and DOSS as outcome indicators and reported conflicting results (Figures 6 and 12). This indicates that the selection of primary outcomes influenced our judgment with regard to curative effects. We selected DOSS as an exploratory outcome since it is not extensively used in dysphagia diagnosis.

## 5. Limitations

This review has various limitations: (i) although most of the included articles used clinical success rate as an outcome indicator, there is no uniform international standard for the definition of success rate. Therefore, we did not consider clinical response rate as one of the outcome indicators, which may have caused a loss of evidence; (ii) poststroke dysphagia is one of the most prevalent stroke complications with high morbidity and a long disease course. The shortest course of treatment in the included studies is 10 days, while the longest is 8 weeks; and only one study [36] had a 3-monthfollow-up and reported a long-term efficacy; and (iii) although we used Egger's test to evaluate publication bias in this review, the fact that all the included RCTs were conducted in China potentially contributes to a publication bias.

## 6. Conclusions

This meta-analysis provided positive evidence that acupuncture or acupuncture combined with rehabilitation were better than using rehabilitation alone in the treatment of poststroke dysphagia. Meanwhile, multicenter RCTs with a large sample and a rigorous design are needed to explore whether acupuncture could replace other stimulative therapies in rehabilitation training. Moreover, acupoint combination, frequency, and the total number of treatments may be important factors that could influence therapeutic effect, which can provide guidance for subsequent similar RCTs.

## Figures and Tables

**Table 1:** The characteristics of the PICO.

References	Sample size (AC + Re/Re) [AC/Re]	Age (year)	Disease duration (d)	Intervention		Intervention time (days)	Outcome
				Treatment	Control		
Chen and Guan [23]	40/40	AC + Re: 62.34 ± 12.53 Re: 64.67 ± 13.42	AC + Re: 450.3 ± 247.8 Re: 489.6 ± 282.3	AC (1/d) + ST + NES	ST + NES (1/d)	14 (5/W)	①⑦
Gao et al. [24]	52/49	AC + Re: 60.25 ± 8.36 Re: 61.37 ± 7.36	1～7	AC (1/d) + NES	NES (2/d)	27∼29 (5-6/W)	①
Jiang et al. [25]	62/58	AC + Re: 60 ± 10 Re: 60 ± 9	AC + Re: 16.46 ± 9.06 Re: 18.97 ± 8.09	AC (1/d) + ST + NES	ST (2/d) + NES	28 (5/W)	⑤⑥⑩⑪
Jing and Jiang [26]	28/29 [28/29]	AC + Re: 62.04 ± 4.77 Re: 60.93 ± 4.56 AC: 61.46 ± 4.53	AC + Re: 70.3 ± 38.08 Re: 73.4 ± 48.53 AC: 52.6 ± 42.39	AC (1/d) + ST + NES	ST (1/d) + NES	28 (5/W)	③
Li et al. [27]	30/30 [30/30]	—	—	AC (1/d) + ST	ST (1/d)	28 (6/W)	①④
Li et al. [28]	40/40	AC + Re: 61.9 ± 7.9 Re: 63.6 ± 6.9	AC + Re: 16.9 ± 7.1 Re: 18.5 ± 8.1	AC (1/d) + ST	ST (2/d)	28 (6/W)	③
Liang et al. [29]	52/50	AC + Re: 56.06 ± 8.15 Re: 54.34 ± 7.72	AC + Re: 40.94 ± 36.01 Re: 43.46 ± 39.43	AC (1/d) + ST	ST (1/d)	21 (6/W)	①
Xiaoping et al. [30]	48/49	AC + Re: 67.0 ± 10.8 Re: 67.1 ± 10.5	AC + Re: 41.1 ± 38.6 Re: 40.5 ± 30.8	AC (1/d) + ST	ST (1/d)	56 (5/W)	①③⑨
Song [31]	30/30	AC + Re: 60.72 ± 8.30 Re: 61.62 ± 8.06	AC + Re: 64.8 ± 31.5 Re: 62.7 ± 40.8	AC (3/w) + ST	ST (1/d)	28 (AC: 3/W; R:5/W)	①④
Wang et al. [32]	45/45	AC + Re: 65 ± 4 Re: 66 ± 4	AC + Re: 1324.95 ± 708.1 Re: 1168 ± 631.45	AC (2/d) + ST	ST (2/d)	21 (5/W)	②⑥
Wang and Shen [33]	30/30	AC + Re: 55.86 ± 8.93 Re: 56.12 ± 9.04	AC + Re: 64.09 ± 10.51/Re: 63.28 ± 10.35	AC (3/w) + ST	ST (1/d)	42 (AC: 3/W; R: 5/W)	①④⑨
Wang [34]	50/50	AC + Re: 57.84 ± 5.25 Re: 60.27 ± 6.32	AC + Re: 56.4 ± 10.8 Re: 52.8 ± 6.9	AC (1/d) + ST	ST (1/d)	10 (1/d)	④
Wu [35]	30/30	AC + Re: 63.76 ± 9.46 Re: 63.72 ± 9.24	AC + Re: 35.12 ± 12.50 Re: 34.76 ± 12.74	AC (1/d) + ST	ST (1/d)	42 (5/W)	①②⑤
Xia et al. [36]	60/60	AC + Re: 65.3 ± 14.2 Re: 66.1 ± 14.3	AC + Re: 9.3 ± 2.3 Re: 8.7 ± 2.5	AC (1/d) + ST	ST (1/d)	28 (6/W)	③⑦⑧⑨
Xing et al. [37]	49/48	AC + Re: 66.9 ± 7.3 Re: 67 ± 7.2	AC + Re: 28.1 ± 3.5 Re: 28.1 ± 3.4	AC (1/d) + ST	ST (1/d)	28 (AC: 5/WR: 7/w)	②③
Xu [38]	20/20 [20/20]	AC + Re: 64.05 ± 10.27 Re: 67.4 ± 8.78 AC: 61.5 ± 7.16	—	AC (1/d) + ST	ST	28 (6/W)	①
Yang et al. [39]	20/20	AC + Re: 61.90 ± 10.30/Re: 62.70 ± 10.10	AC + Re: 75.90 ± 25.50 Re: 79.10 ± 15.10	AC (1/d) + ST	ST (1/d)	14 (6/W)	②
Zheng and Sun [40]	43/42	AC + Re: 62.57 ± 9.77/Re: 61.26 ± 9.59	AC + Re: 23.06 ± 6.91 Re: 22.72 ± 6.56	AC (1/d) + ST	ST (1/d)	28 (6/w)	②③⑥⑧
Zhou et al. [41]	60/50	AC + Re: 59.4 ± 2.6/Re: 58.3 ± 3.1	14∼182	AC (1/d) + ST	ST (1/d)	28 (1/d)	①
Zhou et al. [42]	31/30	AC + Re: 59.90 ± 3.87 Re: 60.43 ± 4.07	AC + Re: 34.81 ± 12.02 Re: 29.30 ± 9.87	AC (1/d) + ST + NES	ST + NES (1/d)	28 (6/w)	③⑨
Xie [43]	[38/38]	AC: 55.53 ± 13.91 Re: 58.95 ± 13.44	AC: 59.66 ± 79.52 Re: 65.05 ± 105.64	AC (1/d)	NES (1/d)	20 (1/d)	①
Duan and Wang [44]	[25/25]	AC: 64.4 ± 7.28 Re: 64.96 ± 7.52	AC: 50.48 ± 16.28 Re: 57.60 ± 17.76	AC (1/d)	ST (1/d)	28 (6/w)	①③
Wang et al. [45]	[45/45]	AC: 65.32 ± 7.24 Re: 65.73 ± 6.25	AC: 26.85 ± 2.27 Re: 26.12 ± 3.31	AC (2/d)	ST (2/d)	28 (6/w)	③
Yang et al. [46]	[34/32]	AC: 62.11 Re: 61.56	15∼90	AC (1/d)	ST + NES (1/d)	30 (1/d)	①
Yu et al. [47]	21/21	AC + Re: 71 ± 7 Re: 71 ± 6	AC + Re: 62.02 ± 33.6 Re: 65.03 ± 42.7	AC (1/d) + ST + NES	ST + NES (1/d)	21 (5/W)	①⑨
Tian et al. [50]	33/32	AC + Re: 57.13 ± 1.62 Re: 57.15 ± 1.59	AC + Re: 23.41 ± 4.73 Re: 23.45 ± 4.71	AC (1/d) + ST + NES	ST (1/d) + NES	28 (5/W)	③⑨⑩⑪
Li et al. [52]	62/62	AC + Re: 65.7 ± 5.2 Re: 64.2 ± 5.7	AC + Re: 2.25 ± 0.92 Re: 2.41 ± 0.83	AC (1/d) + ST	ST (1/d)	28 (5/W)	①⑨
Shen et al. [55]	30/30	AC + Re: 65.23 ± 11.13 Re: 64.76 ± 11.51	AC + Re: 41.4 ± 39.6 Re: 40.5 ± 33.3	AC (1/d) + ST	ST (1/d)	28 (6/W)	①②③⑨
Huang et al. [53]	32/32	AC + Re: 66.00 (62.50, 72.50) Re: 65.00 (57.50, 76.50)	AC + Re: 180 (14.7, 315) Re: 60 (4.2, 330)	AC (1/d) + ST	ST (1/d)	28 (3/W)	①
Gao and Zhou [56]	42/42	AC + Re: 62.95 ± 8.99 Re: 62.43 ± 10.12	AC + Re: 55.2 ± 62.1 Re: 30.9 ± 22.8	AC (1/d) + ST + NES	NES + ST (1/d)	28 (5/W)	①③⑥⑩⑪
Wang et al. [49]	38/38	AC + Re: 67.0 ± 10.8 Re: 67.1 ± 10.5	AC + Re: 5.94 ± 6.81 Re: 6.32 ± 2.56	AC (1/d) + NES + ST	NES + ST (1/d)	21 (6/W)	①②⑨
Wang [48]	33/32	AC + Re: 63.58 ± 10.288 Re: 63.90 ± 10.189	AC + Re: 41.32 ± 37.01 Re: 36.06 ± 37.73	AC (6/w) + NES + ST	ST (1/d)	28 (6/W)	②③
Qi et al. [51]	60/60	AC + Re: 63 ± 10 Re: 63 ± 11	AC + Re: 14.2 ± 4.1 Re: 15.2 ± 3.8	AC (1/d) + ST	ST (1/d)	14 (7/W)	②③

AC: acupuncture; Re: rehabilitation; ST: swallowing training; NES: neuromuscular electrical stimulation. *Note*. means acupuncture alone group compared with rehabilitation alone group; ① WST. ② VFSS. ③ SSA scores. ④ Swallowing scores of Fujishima Ichiro. ⑤ The rates of aspiration. ⑥ The rates of aspiration pneumonia. ⑦ DOSS. ⑧ BI. ⑨ SWAL-QOL. ⑩ Duration of empty swallowing⑪ Duration of 5 mL water swallowing.

**Table 2:** The characteristics of manual acupuncture.

References	Acupoints	Reinforcing and reducing	Needle retaining time
Chen and Guan [23]	Three tongue needle	Mild supplementing and reducing	30 min
Gao et al. [24]	Three tongue needle	Mild supplementing and reducing	30 min
Jiang et al. [25]	Tongue acupuncture: heart point, spleen point, and kidney point	—	0 min
Jing and Jiang [26]	Lianquan (CV23), Panglianquan, Shanglianquan, Yifeng (TE17), Fengchi (GB20), and Jingbailao (EX-HN15)	Mild supplementing and reducing	30 min
Li et al. [27]	Fengchi (GB20), Lianquan (CV23), Shanglianquan, Jinjing (EX-HN12), Yuye (EX-HN13), Lieque (LU7), Dicang (ST41), Jiache (ST6), Xiaguan (ST7), and Jiachengjiang	GB20 (reinforcing)	30 min
Li et al. [28]	Fengchi (GB20), Yiming (EX-HN14), Gongxuepoint, Zhiqiangpoint, Tunyanpoint, Lianquan (CV23), Waijinjin, and Waiyuye	GB20, EX-HN14 and Gongxuepoint retaining needle, others not	30 min
Liang et al. [29]	Scalp motor area low 2/5, Fengchi (GB20), Yiming (EX-HN14), Gongxuepoint, Zhiqiangpoint, Tunyanpoint, Lianquan (CV23), Waijinjin, Waiyuye Yifeng (TE17), Qianzheng, Yingxiang (LI20), Jiachengjiang, scalp emotional area, foot motor sensory area, Shenshu (BL23), Huiyang (BL35), Xiaguan (ST7), Jiache (ST6), Sizhukong (TE23), Fayinpoint, and Fanliupoint	—	30 min
Xiaoping et al. [30]	Fengchi (GB20), Yiming (EX-HN14), Gongxue point, Lianquan (CV23), Jinjing (EX-HN12), Yuye (EX-HN13), Tunyan point, Zhiqiang point, and Fayin point	GB20, EX-HN14 and Gongxuepoint retaining needle, others not	30 min
Song [31]	Tiantu (CV22)	—	20–30 min
Wang et al. [32]	Aqiang point, Zhiqiang point, Tunyan point, Tiiyan point, and Fayin point	—	30 min
Wang and Shen [33]	Baihui (GV20),Sishencong (EX-HN1), language area, Lianquan (CV23), Jinjing (EX-HN12), and Yuye (EX-HN13)	—	30 min
Wang [34]	Fengchi (GB20), Yifeng (TE17), Tiantu (CV22), and piercing the pharynx posterior wall	—	—
Wu [35]	Taixi (KI3), Fengchi (GB20), Lianquan (CV23), Jialianquan, Jinjing (EX-HN12), Yuye (EX-HN13), and pharynx posterior wall	—	30 min
Xia et al. [36]	Fengchi (GB 20, unilateral), Jiaji (C2–C4) (EX-B2, bilateral), Lianquan (CV 23, unilateral), Jiajianquan (left CV 23 and right CV 23, bilateral), Baihui (GV 20, unilateral), Lieque (LV 07, bilateral), Fenglong (ST 40, bilateral), Sanyinjiao (SP 06, bilateral), Jinjin (EX-HN 12, unilateral), Yuye (EX-HN13, unilateral), Taixi (K 103, bilateral), and Zhaohai (K 106, bilateral)	PC6, HT1, LU5, BL40, LR3, ST40, LI1, ST44, LI4 reducing, SP6, KI3, CV6, ST36 reinforcing GV20, GB20, CV23 mild supplementing and reducing piercing EX-HN12, and EX-HN13	30 min
Xing et al. [37]	Dazhui (GV14), Fengfu (GV16), Shenting (GV24), Shendao (GV11), Baihui (GV20), Shuigou (GV26), Qimen (LR14), Danzhong (CV17), Shenshu (BL23), Ganshu (BL18), Sanyinjiao (SP6), Xinshu (BL15), Pishu (BL20), Tiantu (CV22), Yinlingquan (SP9), Lianquan (CV23), Fenglong (ST40), Waiguan (TE5), Xiaxi (GB43), and Xingjian (LR2)	—	30 min
Xu [38]	Tongue acupuncture: heart point and Lianquan (CV23)	—	30 min
Yang et al. [39]	Lianquan (CV23) and Fengchi (GB20)	GB20 reinforcing	30 min
Zheng and Sun [40]	Liangquan (CV23), Jinjing (EX-HN12), Yuye (EX-HN13), Fengchi (GB20), Yifeng (TE17), Waiguan (TE5), Quchi (LI11), Binao (LI14), Yongquan (KI1), Zusanli (ST36), and Siqiang	GB20 reinforcing TE17, CV23 mild supplementing and reducing piercing EX-HN12, and EX-HN13	—
Zhou et al. [41]	Aqiang point: Aqiang point, Zhiqiang point, Tunyan point, Tiyan point, Fayin point	—	30 min
Zhou et al. [42]	MS6 low 2/5 and MS10	—	20 min
Xie [43]	Three tongue needles	Lifting and thrusting until “deqi” then mild supplementing and reducing	30 min
Duan and Wang [44]	Tianrong (SI17, bilateral), Lianquan (CV23), Waijinjin, and Waiyuye	SI17 (twisting reducing)	30 min
Wang et al. [45]	Aqiang point, Tunyan point, and Tiyan point	Twirling, lifting, and thrusting slowly	20 min
Yang et al. [46]	Lianquan (CV23), Fengchi (GB20), Yifeng (TE17), and Lieque (LU7)	—	30 min
Yu et al. [47]	The bottom 2/5 in the anterior parietal temporal oblique and posterior parietal oblique, Fengchi (GB20), Yiming (EX-HN14), Gongxuepoint, Zhiqiangpoint, Tunyanpoint, Fayinpoint, Lianquan (CV23), Waijinjin, and Waiyuye	GB20, EX-HN14 and Gongxuepoint twirling and retaining, others not	30 min
Tian et al. [50]	Lianquan (CV23), Panglianquan, Fengchi (GB20), Wangu (GB12), Yifeng (TE 17), Jinjin (EX-HN 12), Yuye (EX-HN13)	Twirling	30 min
Li et al. [52]	Neiguan (PC6), Shuigou (GV26), Sanyinjiao (SP6), Fengchi (GB20), Wangu (GB12), Yifeng (TE 17), pharynx posterior wall, and Lianquan (CV23)	PC6 (reducing by lifting and thrusting with twirling), GV26 (reducing by bird-peck needling), SP6 (reinforcing by twirling), GB20, GB12, TE 17 (reinforcing by twirling), and CV23 (reducing by twirling)	30 min
Shen et al. [55]	Lianquan (CV23), Pang Lianquan, Jinjin (EX-HN 12), and Yuye (EX-HN13)	CV23 (lifting and thrusting) and CV23 (lifting and thrusting with twirling)	30 min
Huang et al. [53]	Baihui (GV20), Fengchi (GB20), Fengfu (GV16), Yamen (GV15), Yifeng (TE17), Lianquan (CV23), Jinjin (EX-HN12), Yuye (EX-HN13), and Zusanli (ST36)	—	30 min
Gao and Zhou [56]	Fengchi (GB20), Tianzhu (BL10), Wangu (GB12), Lianquan (CV23), Panglianquan, Jinjin (EX-HN 12), and Yuye (EX-HN13)	—	30 min
Wang et al. [49]	Shexia, Fengchi (GB20), Wangu (GB12), Tianzhu (BL10), and Yifeng (TE17)	GB20, GB12, BL10, BL10, and TE17 : High frequency and small amplitude	30 min
Wang [48]	Fengfu (GV16), Fengchi (GB20), Yifeng (TE 17), Lianquan (CV23), Liyanpoint1, Liyanpoint2, Shenmen (HT7), Lieque (LU7), and Zhaohai (LI6)	—	30 min
Qi et al. [51]	Fengchi (GB20), Tianzhu (BL10), Wangu (GB12), Lianquan (CV23), Panglianquan, Jinjin (EX-HN 12), and Yuye (EX-HN13),	GB20, BL10, GB12, CV23, and PangLianquan (twirling)	30 min

**Table 3:** The characteristics of the rehabilitation training.

References	Content
Chen and Guan [23]	Viralstim type low-frequency pulse electrical stimulation and direct and indirect strategies
Gao et al. [24]	Swallowing disorder therapeutic apparatus
Jiang et al. [25]	Rehabilitation training + low-frequency pulse electrical
Jing and Jiang [26]	Low-frequency neuromuscular electrical stimulation, ice stimulation + speech training + lip reduction training + practice blowing or whistling + chewing training + lip exercise training + empty swallowing action
Li et al. [27]	Swallowing function training, ice stimulation compensation strategy:(1) the Mendelson's technique, (2) supraglottic swallowing, (3) nodding swallowing, and (4) turn the head and swallowing, and direct strategy: food
Li et al. [28]	Oral sensorimotor training, Shaker training, Masako training, and Mendelsohn training
Liang et al. [29]	Strength training, exercise relearning, biofeedback, temperature tactile stimulation, and swallowing manipulation therapy
Xiaoping et al. [30]	Indirect training + direct training indirect training methods: (a) swallowing muscle training, (b) pressing exercises, (c) closed glottis training, (d) Mendelsohn maneuver, (e) ice stimulation, and (f) the tongue muscle training direct training method: (a) food placement, (b) food form, (c) feeding posture, (d) gradually adjusting food intake, and (e) interactive swallowing
Song [31]	Indirect training: cheek, lip and other swallowing muscle training, tongue muscle training, vocal cord closure training, cold stimulation, breath-holding and vocal exercise, the Mendelsohn method, and supraglottic swallowing direct training: posture when eating, food shape, and bitesize
Wang et al. [32]	Swallowing muscle training, tongue muscle training, vocal cord closure training, swallowing reflex training, buccinator and tongue muscle training, mandibular and tongue exercise training, ice stimulation, posture adjustment, and removal of retained food in the pharynx, etc.
Wang and Shen [33]	Swallowing rehabilitation training such as tongue training, lip training, and eating training
Wang [34]	Basic and feeding training. Basic rehabilitation training: cold stimulation of the pharynx, lip movement rehabilitation training, tongue movement rehabilitation training, jaw movement rehabilitation training ingestion rehabilitation training.
Wu [35]	Rehabilitation training for swallowing disorders: breathing training, neck mobility training, exercise training around the oral cavity and tongue muscles, pharynx ice stimulation, and Shaq (training, swallowing mode training, and Mendelsohn training)
Xia et al. [36]	Functional training active or passive exercise of the oral, facial, and lingual muscles, and sensory stimuli (containing the Mendelsohn maneuver, supraglottic and supraglottic maneuvers, swallowing efforts, and the Shaker exercise)
Xing et al. [37]	Tongue muscle exercise, buccal muscle exercise, the breathing exercise method, and the throat muscle exercise method
Xu [38]	Ingestion training + swallowing function training + ice stimulation + cough training + neck rotation training
Yang et al. [39]	Basic training and direct feeding training basic training: ice stimulation, tongue exercise training, the Mendelsson technique training, chin exercise training, breathing and cough training, lip and cheek exercise training, pronunciation, and vocal cord adduction training, etc. Direct feeding training: diet training, pharyngeal food removal training, swallowing skills practice, and breath-holding training
Zheng and Sun [40]	Ice stimulation, tongue movement training, soft palate lift training, throat movement training, and eating training
Zhou et al. [41]	Swallowing training: tongue, jaw, upper, and lower lips
Zhou et al. [42]	Rehabilitation training + vocastim-maste swallowing disorder therapeutic apparatus
Xie [43]	Swallowing disorder therapeutic apparatus
Duan and Wang [44]	Swallowing organ training, ice stimulation, and eating training
Wang et al. [45]	Swallowing muscle training, tongue muscle training, vocal cord closure training, swallowing reflex training, mandibular and tongue exercise training, and ice stimulation
Yang et al. [46]	Swallowing disorder therapeutic apparatus, ice stimulation
Yu et al. [47]	Direct strategy: food Indirect swallowing training: pronunciation training, tongue muscle and masticatory muscle training, sucking training, laryngeal lift training, and glottis atresia training neuromuscular electrical stimulation
Tian et al. [50]	Indirect training includes sensory stimulation and oral motor training, such as ice stimulation, lip movement, jaw movement, and tongue movement training. Direct training includes feeding training, including eating utensils, food selection, eating position, eating environment, swallowing methods, acupuncture swallowing, onsonic swallowing, interactive swallowing and Myo trac dual-channel biostimulation feedback device
Li et al. [52]	Breathing training treatment includes abdominal breathing training, pursed lip breathing training, and active circuit breathing training practice
Shen et al. [55]	Feeding training, swallowing reflex training, and tongue training
Huang et al. [53]	Supraglottic swallowing, the Mendelsohn method, the Shaker method, tongue exercise, and orofacial myofunctional exercises, cold stimulation to oral cavity and throat, and vocal cord closure exercise
Gao and Zhou [56]	Lip atresia training, tongue muscle training, jaw exercise training, ice stimulation training, breath-holding training, Mendelson's technique, and low-frequency neuromuscular electrical stimulation
Wang et al. [49]	Oral neuromuscular training, facial sensory vibration, and compensatory techniques, etc.
Wang [48]	Ice stimulation, Shaker training, Mendelson's technique, breathing training, and neuromuscular electrical stimulation
Qi et al. [51]	Swallowing organ exercise training, vocalization exercise, gag reflex training, tongue muscle function training, and feeding training respiratory function training: deep breathing training, narrow mouth breathing training, abdominal relaxation training, diaphragm activity training, and abdominal breathing

**Table 4:** Evidence quality evaluation.

Certainty assessment							No. of patients		Effect (95% CL)	Certainty
No of studies	Reference no	Risk of bias	Inconsistency	Indirectness	Imprecision	Publication bias	Acupuncture Group	Rehabilitation Group		
*Manual acupuncture compared to rehabilitation (WST)*										
5	Randomized trials	Serious	No serious inconsistency	No serious indirectness	No serious imprecision	Strongly suspected	147	145	MD 0.47 lower (0.72 to 0.23 lower)	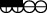 Low

*Manual acupuncture combined with rehabilitation compared to rehabilitation (WST)*										
16	Randomized trials	Serious	No serious inconsistency	No serious indirectness	No serious imprecision	None	618	602	MD 0.74 lower (0.96 to 0.52 lower)	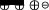 Moderate

*Manual acupuncture compared to rehabilitation(VFSS)*										
9	Randomized trials	Serious	No serious inconsistency	No serious indirectness	No serious imprecision	None	346	344	MD 1.35 higher (1.00 to 1.71 higher)	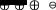 Moderate

## Data Availability

The datasets generated during and analyzed during the current study are available from the corresponding author on reasonable request.

## Supplementary Materials

Supplementary Materials Table S1: search strategy. Table S2: PRISMA-P 2020 Checklist. Figure S3: the sensitivity analysis of WST of acupuncture alone. Figure S4: the sensitivity analysis of SSA of acupuncture alone. Figure S5: the sensitivity analysis of WST of acupuncture combined with rehabilitation. Figure S6: the sensitivity analysis of VFSS of acupuncture combined with rehabilitation. Figure S7: the sensitivity analysis of SSA of acupuncture combined with rehabilitation. Figure S8: the sensitivity analysis of swallowing scores of Fujishima Ichiro of acupuncture combined with rehabilitation. Figure S9: the sensitivity analysis of the rates of aspiration of acupuncture combined with rehabilitation. Figure S10: the sensitivity analysis of the rates of aspiration pneumonia of acupuncture combined with rehabilitation. Figure S11: the sensitivity analysis of DOSS of acupuncture combined with rehabilitation. Figure S12: the sensitivity analysis of BI of acupuncture combined with rehabilitation. Figure S13: the sensitivity analysis of SWAL-QOL of acupuncture combined with rehabilitation. Figure S14: the sensitivity analysis of duration of empty swallowing (submental muscle group) of acupuncture combined with rehabilitation. Figure S15: the sensitivity analysis of duration of empty swallowing (infrahyoid muscles) of acupuncture combined with rehabilitation. Figure S16: the sensitivity analysis of duration of 5 mL water swallowing (submental muscle group) of acupuncture combined with rehabilitation. Figure S17: the sensitivity analysis of duration of 5 mL water swallowing (infrahyoid muscles) of acupuncture combined with rehabilitation.
