# Supplementary material for: Manual Acupuncture or Combination of Rehabilitation Therapy to Treat Poststroke Dysphagia: A Systematic Review and Meta-Analysis of Randomized Controlled Trials
Source: Evid Based Complement Alternat Med. 2022 Oct 15;2022:8803507. doi: 10.1155/2022/8803507 (PMC9588332; doi:10.1155/2022/8803507)
Supplement: Supplementary Materials — Table S1: search strategy. Table S2: PRISMA-P 2020 Checklist. Figure S3: the sensitivity analysis of WST of acupuncture alone. Figure S4: the sensitivity analysis of SSA of acupuncture alone. Figure S5: the sensitivity analysis of WST of acupuncture combined with rehabilitation. Figure S6: the sensitivity analysis of VFSS of acupuncture combined with rehabilitation. Figure S7: the sensitivity analysis of SSA of acupuncture combined with rehabilitation. Figure S8: the sensitivity analysis of swallowing scores of Fujishima Ichiro of acupuncture combined with rehabilitation. Figure S9: the sensitivity analysis of the rates of aspiration of acupuncture combined with rehabilitation. Figure S10: the sensitivity analysis of the rates of aspiration pneumonia of acupuncture combined with rehabilitation. Figure S11: the sensitivity analysis of DOSS of acupuncture combined with rehabilitation. Figure S12: the sensitivity analysis of BI of acupuncture combined with rehabilitation. Figure S13: the sensitivity analysis of SWAL-QOL of acupuncture combined with rehabilitation. Figure S14: the sensitivity analysis of duration of empty swallowing (submental muscle group) of acupuncture combined with rehabilitation. Figure S15: the sensitivity analysis of duration of empty swallowing (infrahyoid muscles) of acupuncture combined with rehabilitation. Figure S16: the sensitivity analysis of duration of 5 mL water swallowing (submental muscle group) of acupuncture combined with rehabilitation. Figure S17: the sensitivity analysis of duration of 5 mL water swallowing (infrahyoid muscles) of acupuncture combined with rehabilitation. [file 8803507.f1.zip › Table S1 Search strategy (1).docx]

Pubmed .....................................................38

#6.

Search: #1 AND #2 AND #3 AND #4 AND #5

****((((stroke∗OR Poststroke OR Cerebrovascular OR CVA∗ OR Apoplexy OR Vascular Accident∗ OR brain OR Cerebral∗) AND (Point∗OR Acupuncture OR Acupoint∗)) AND (Swallowing Disorder∗OR Dysphagia OR Deglutition Disorder∗)) AND (Randomized OR RCT OR Randomly)) AND (Trial∗)****

[38](https://pubmed.ncbi.nlm.nih.gov/?term=((((stroke%E2%88%97OR+Poststroke+OR+Cerebrovascular+OR+CVA%E2%88%97+OR+Apoplexy+OR+Vascular+Accident%E2%88%97+OR+brain+OR+Cerebral%E2%88%97)+AND+(Point%E2%88%97OR+Acupuncture+OR+Acupoint%E2%88%97))+AND+(Swallowing+Disorder%E2%88%97OR+Dysphagia+OR+Deglutition+Disorder%E2%88%97))+AND+(Randomized+OR+RCT+OR+Randomly))+AND+(Trial%E2%88%97)&ac=no&sort=relevance)

#5.

Search: ****Trial∗****

[1,973,276](https://pubmed.ncbi.nlm.nih.gov/?term=Trial%E2%88%97&ac=no&sort=relevance)

#4.

Search: ****Randomized OR RCT OR Randomly****

[1,591,596](https://pubmed.ncbi.nlm.nih.gov/?term=Randomized+OR+RCT+OR+Randomly&ac=no&sort=relevance)

#3.

Search: ****Swallowing Disorder∗OR Dysphagia OR Deglutition Disorder∗****

[58,997](https://pubmed.ncbi.nlm.nih.gov/?term=Swallowing+Disorder%E2%88%97OR+Dysphagia+OR+Deglutition+Disorder%E2%88%97&ac=no&sort=relevance)

#2.

Search: ****Point∗OR Acupuncture OR Acupoint∗****

[13,014](https://pubmed.ncbi.nlm.nih.gov/?term=Point%E2%88%97OR+Acupuncture+OR+Acupoint%E2%88%97&ac=no&sort=relevance)

#1.

Search: ****stroke∗OR Poststroke OR Cerebrovascular OR CVA∗ OR Apoplexy OR Vascular Accident∗ OR brain OR Cerebral∗****

[2,688,030](https://pubmed.ncbi.nlm.nih.gov/?term=stroke%E2%88%97OR+Poststroke+OR+Cerebrovascular+OR+CVA%E2%88%97+OR+Apoplexy+OR+Vascular+Accident%E2%88%97+OR+brain+OR+Cerebral%E2%88%97&ac=no&sort=relevance)

Embase..........................................................82

Session Results

.......................................................

No. Query Results Results Date

#5. #1 AND #2 AND #3 AND #4 82 18 Aug 2022

#4. 'randomized controlled trial':ab,ti OR 'clinical 2,043,036 18 Aug 2022

study':ab,ti OR trials:ab,ti OR rct:ab,ti OR

randomly:ab,ti OR randomized:ab,ti

#3. 'disorder, deglutition':ab,ti OR 'swallowing, 82,427 18 Aug 2022

disorder':ab,ti OR dysphagia:ab,ti OR

oropharyngeal:ab,ti OR 'dysphagia,

esophageal':ab,ti

#2. stroke:ab,ti OR cva*:ab,ti OR apoplexy:ab,ti OR 2,172,350 18 Aug 2022

cerebrovascular:ab,ti OR 'vascular

accident':ab,ti OR brain:ab,ti OR cerebral:ab,ti

#1. acupuncture:ab,ti OR acupoint:ab,ti OR 'point, 38,417 18 Aug 2022

acupuncture':ab,ti

.......................................................

Web of science ....................................................125


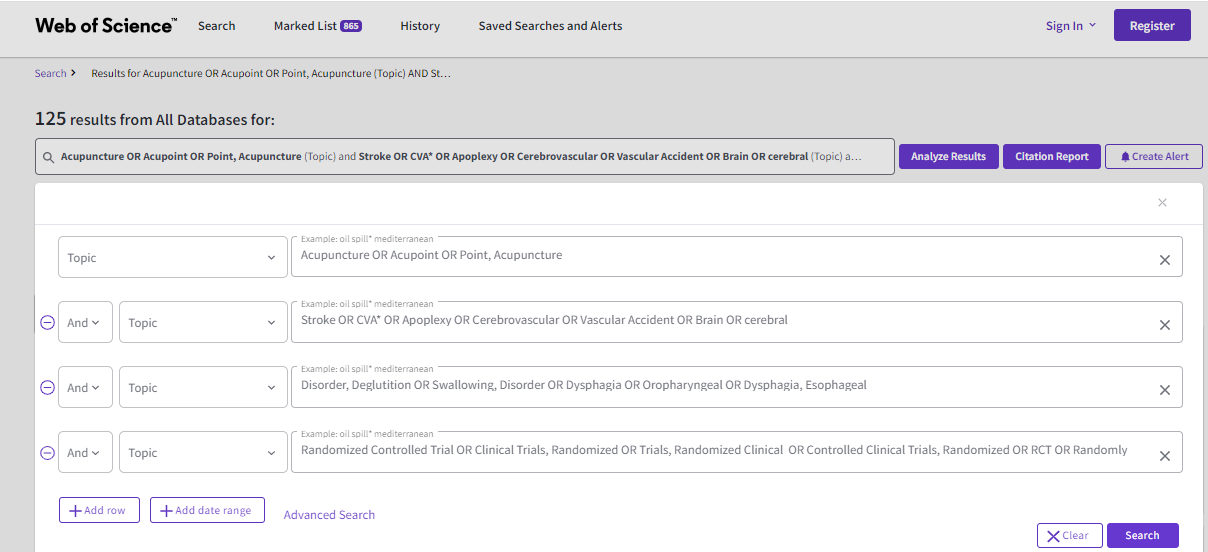


#1 Acupuncture OR Acupoint OR Point, Acupuncture

#2 Stroke OR CVA* OR Apoplexy OR Cerebrovascular OR Vascular Accident OR Brain OR cerebral

#3 Disorder, Deglutition OR Swallowing, Disorder OR Dysphagia OR Oropharyngeal OR Dysphagia, Esophageal

#4 [Randomized Controlled Trial](javascript:;) OR Clinical Trials, Randomized OR Trials, Randomized Clinical OR Controlled Clinical Trials, Randomized OR RCT OR Randomly

# 5 #1AND# 2 AND# 3 AND# 4

Medline ..........................................................45


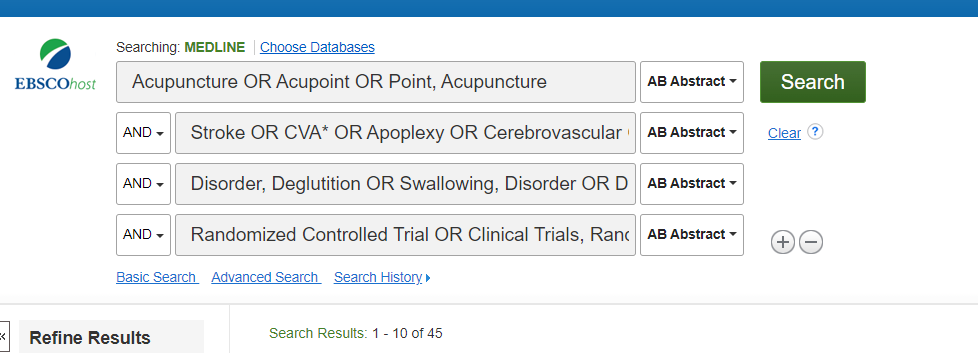


#1 Acupuncture OR Acupoint OR Point, Acupuncture

#2 Stroke OR CVA* OR Apoplexy OR Cerebrovascular OR Vascular Accident OR Brain OR cerebral

#3 Disorder, Deglutition OR Swallowing, Disorder OR Dysphagia OR Oropharyngeal OR Dysphagia, Esophageal

#4 [Randomized Controlled Trial](javascript:;) OR Clinical Trials, Randomized OR Trials, Randomized Clinical OR Controlled Clinical Trials, Randomized OR RCT OR Randomly

# 5 #1AND# 2 AND# 3 AND# 4

Sinomed........................................................ 924


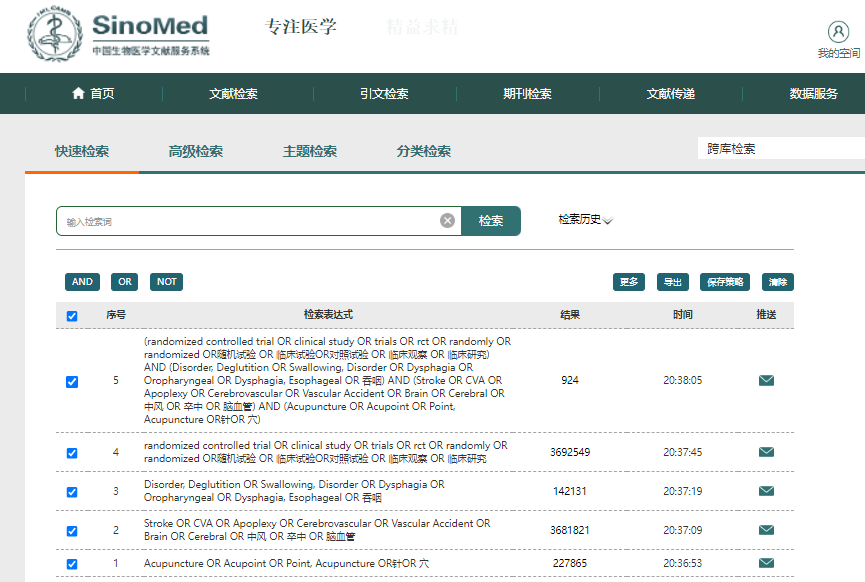


1. #1 Acupuncture OR Acupoint OR Point, Acupuncture OR针OR 穴

227865 2022-08-18 20:36:53.0

1. #2 Stroke OR CVA OR Apoplexy OR Cerebrovascular OR Vascular Accident OR Brain OR Cerebral OR 中风 OR 卒中 OR 脑血管

3681821 2022-08-18 20:37:09.0

1. #3 Disorder, Deglutition OR Swallowing, Disorder OR Dysphagia OR Oropharyngeal OR Dysphagia, Esophageal OR 吞咽

142131 2022-08-18 20:37:19.0

1. #4 randomized controlled trial OR clinical study OR trials OR rct OR randomly OR randomized OR随机试验 OR 临床试验OR对照试验 OR 临床观察 OR 临床研究

3692549 2022-08-18 20:37:45.0

5）# 5 #1AND# 2 AND# 3 AND# 4

(randomized controlled trial OR clinical study OR trials OR rct OR randomly OR randomized OR随机试验 OR 临床试验OR对照试验 OR 临床观察 OR 临床研究) AND (Disorder, Deglutition OR Swallowing, Disorder OR Dysphagia OR Oropharyngeal OR Dysphagia, Esophageal OR 吞咽) AND (Stroke OR CVA OR Apoplexy OR Cerebrovascular OR Vascular Accident OR Brain OR Cerebral OR 中风 OR 卒中 OR 脑血管) AND (Acupuncture OR Acupoint OR Point, Acupuncture OR针OR 穴)

924 2022-08-18 20:38:05.0

Cochrane Library databases ......................................132


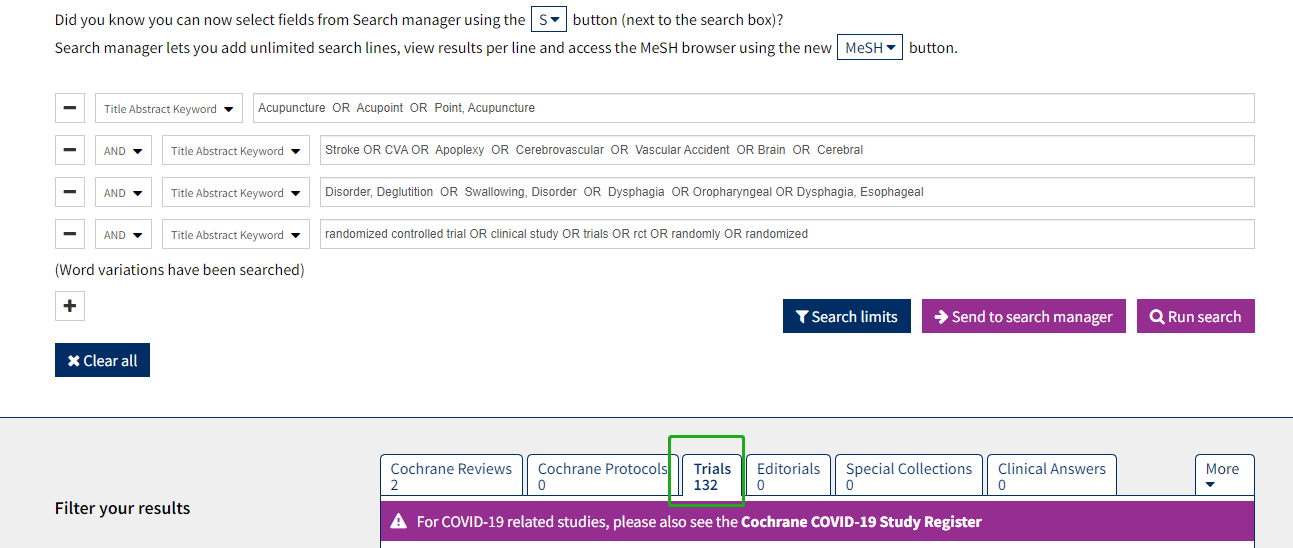


CNKI (China National Knowledge Infrastructure) ................. 748


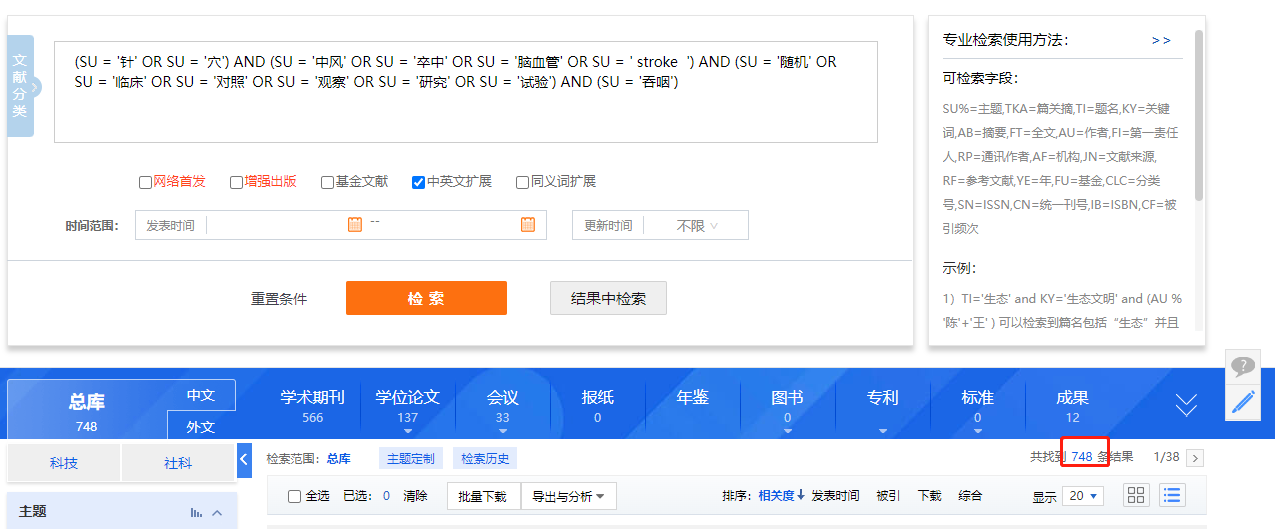


(SU = '针' OR SU = '穴') AND (SU = '中风' OR SU = '卒中' OR SU = '脑血管' OR SU = ' stroke  ') AND (SU = '随机' OR SU = '临床' OR SU = '对照' OR SU = '观察' OR SU = '研究' OR SU = '试验') AND (SU = '吞咽')

WanFang (WanFang Database) ....................................... 2001


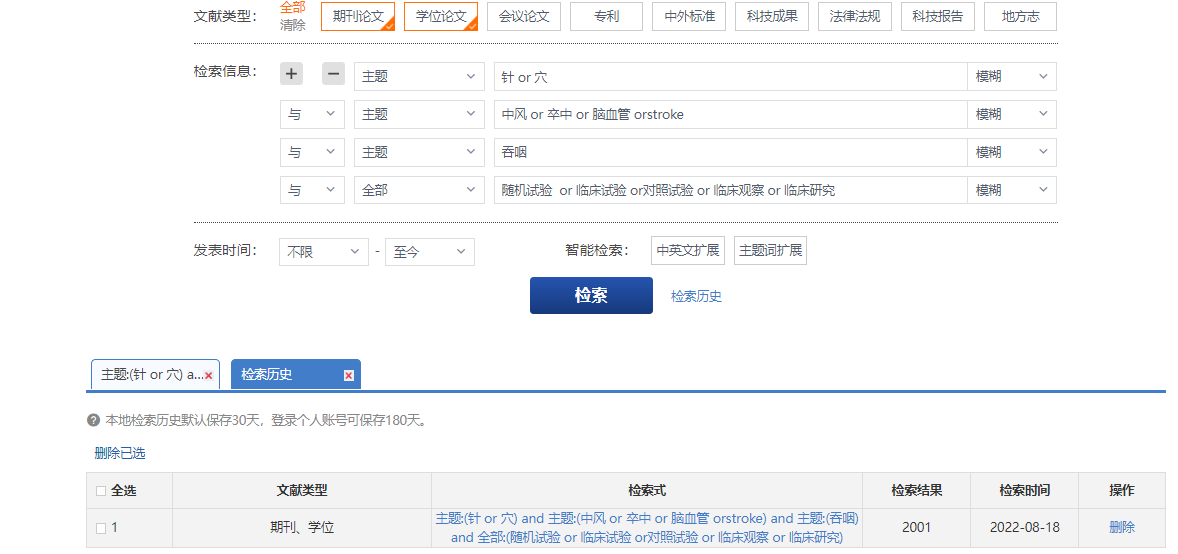


主题:(针 or 穴) and 主题:(中风 or 卒中 or 脑血管 orstroke) and 主题:(吞咽) and 全部:(随机试验 or 临床试验 or对照试验 or 临床观察 or 临床研究)

VIP (Chongqing VIP)..............................................1441


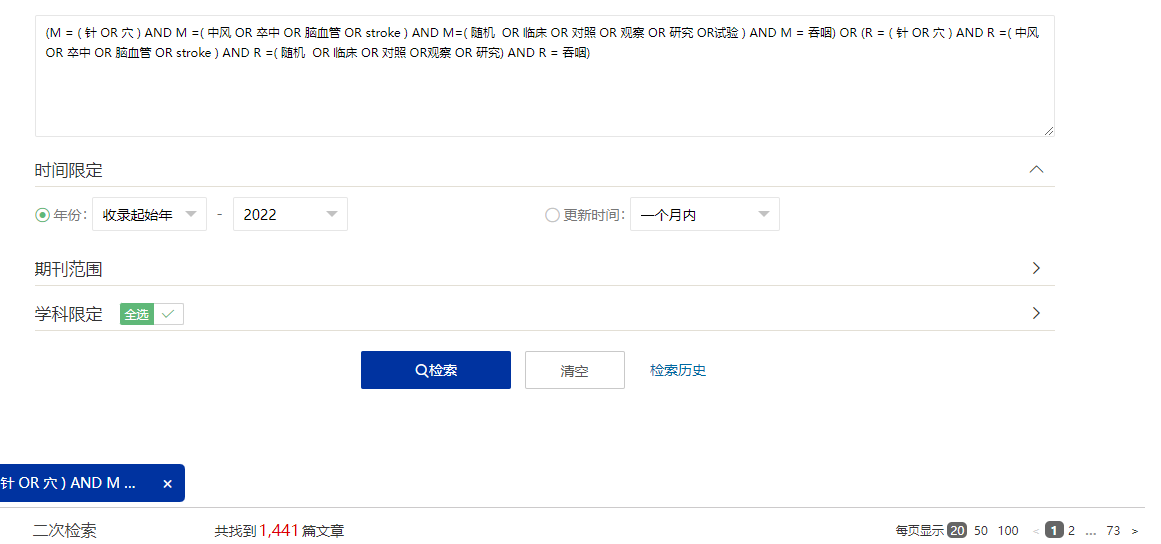


(M = ( 针 OR 穴 ) AND M =( 中风 OR 卒中 OR 脑血管 OR stroke ) AND M=( 随机 OR 临床 OR 对照 OR 观察 OR 研究 OR试验 ) AND M = 吞咽) OR (R = ( 针 OR 穴 ) AND R =( 中风 OR 卒中 OR 脑血管 OR stroke ) AND R =( 随机 OR 临床 OR 对照 OR观察 OR 研究) AND R = 吞咽)
