# Supplementary figures and images for: Manual Acupuncture or Combination of Rehabilitation Therapy to Treat Poststroke Dysphagia: A Systematic Review and Meta-Analysis of Randomized Controlled Trials
Source: Evid Based Complement Alternat Med. 2022 Oct 15;2022:8803507. doi: 10.1155/2022/8803507 (PMC9588332; doi:10.1155/2022/8803507)

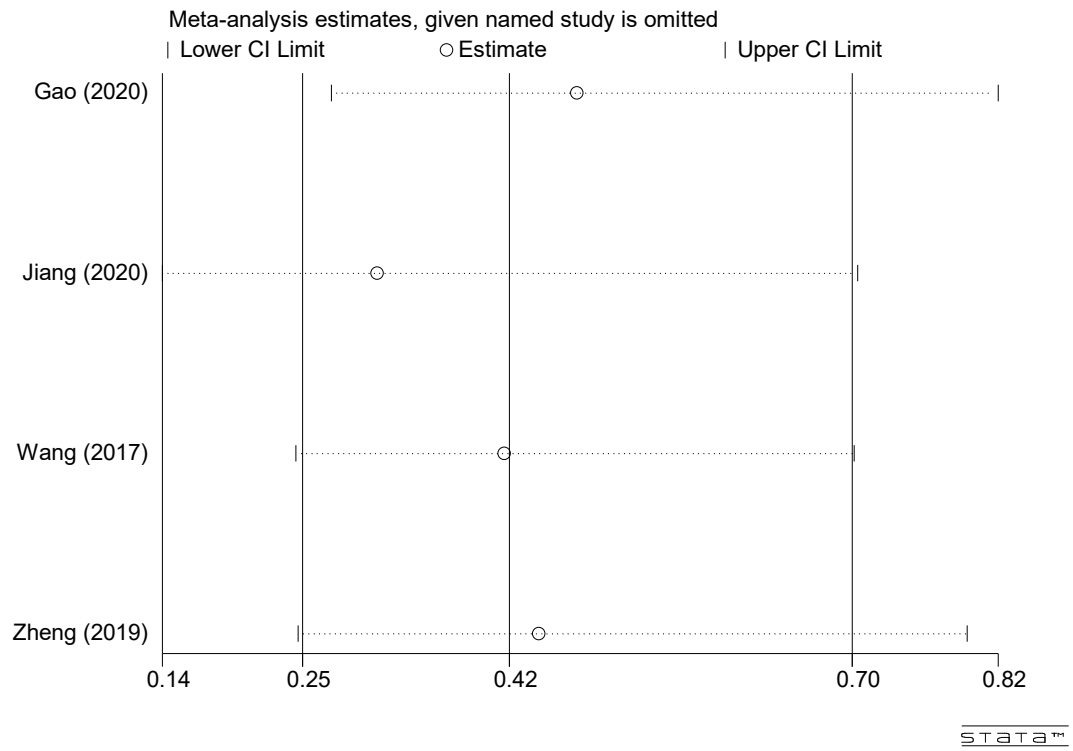

Supplement: Supplementary Materials — Table S1: search strategy. Table S2: PRISMA-P 2020 Checklist. Figure S3: the sensitivity analysis of WST of acupuncture alone. Figure S4: the sensitivity analysis of SSA of acupuncture alone. Figure S5: the sensitivity analysis of WST of acupuncture combined with rehabilitation. Figure S6: the sensitivity analysis of VFSS of acupuncture combined with rehabilitation. Figure S7: the sensitivity analysis of SSA of acupuncture combined with rehabilitation. Figure S8: the sensitivity analysis of swallowing scores of Fujishima Ichiro of acupuncture combined with rehabilitation. Figure S9: the sensitivity analysis of the rates of aspiration of acupuncture combined with rehabilitation. Figure S10: the sensitivity analysis of the rates of aspiration pneumonia of acupuncture combined with rehabilitation. Figure S11: the sensitivity analysis of DOSS of acupuncture combined with rehabilitation. Figure S12: the sensitivity analysis of BI of acupuncture combined with rehabilitation. Figure S13: the sensitivity analysis of SWAL-QOL of acupuncture combined with rehabilitation. Figure S14: the sensitivity analysis of duration of empty swallowing (submental muscle group) of acupuncture combined with rehabilitation. Figure S15: the sensitivity analysis of duration of empty swallowing (infrahyoid muscles) of acupuncture combined with rehabilitation. Figure S16: the sensitivity analysis of duration of 5 mL water swallowing (submental muscle group) of acupuncture combined with rehabilitation. Figure S17: the sensitivity analysis of duration of 5 mL water swallowing (infrahyoid muscles) of acupuncture combined with rehabilitation. [file 8803507.f1.zip › Fig.S10 The sensitivity analysis of The rates ofaspirationpneumonia of acupuncture combine with Rehabilitation (1).pdf]

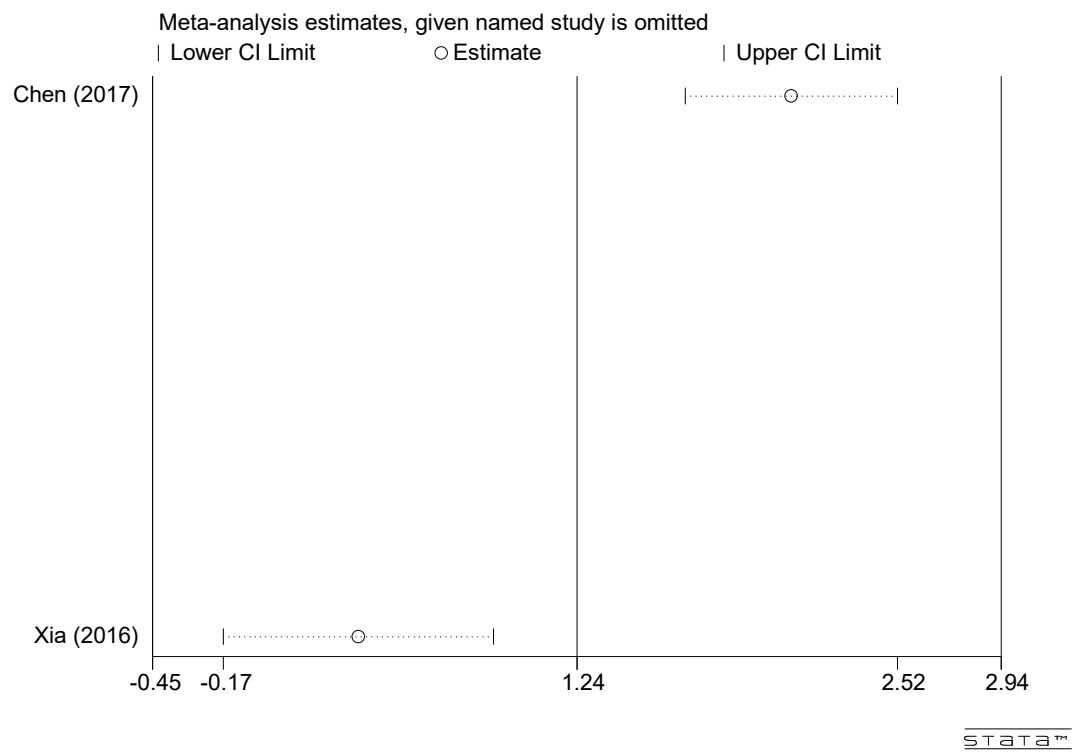

Supplement: Supplementary Materials — Table S1: search strategy. Table S2: PRISMA-P 2020 Checklist. Figure S3: the sensitivity analysis of WST of acupuncture alone. Figure S4: the sensitivity analysis of SSA of acupuncture alone. Figure S5: the sensitivity analysis of WST of acupuncture combined with rehabilitation. Figure S6: the sensitivity analysis of VFSS of acupuncture combined with rehabilitation. Figure S7: the sensitivity analysis of SSA of acupuncture combined with rehabilitation. Figure S8: the sensitivity analysis of swallowing scores of Fujishima Ichiro of acupuncture combined with rehabilitation. Figure S9: the sensitivity analysis of the rates of aspiration of acupuncture combined with rehabilitation. Figure S10: the sensitivity analysis of the rates of aspiration pneumonia of acupuncture combined with rehabilitation. Figure S11: the sensitivity analysis of DOSS of acupuncture combined with rehabilitation. Figure S12: the sensitivity analysis of BI of acupuncture combined with rehabilitation. Figure S13: the sensitivity analysis of SWAL-QOL of acupuncture combined with rehabilitation. Figure S14: the sensitivity analysis of duration of empty swallowing (submental muscle group) of acupuncture combined with rehabilitation. Figure S15: the sensitivity analysis of duration of empty swallowing (infrahyoid muscles) of acupuncture combined with rehabilitation. Figure S16: the sensitivity analysis of duration of 5 mL water swallowing (submental muscle group) of acupuncture combined with rehabilitation. Figure S17: the sensitivity analysis of duration of 5 mL water swallowing (infrahyoid muscles) of acupuncture combined with rehabilitation. [file 8803507.f1.zip › Fig.S11 The sensitivity analysis of DOSS of acupuncture combine with Rehabilitation.pdf]

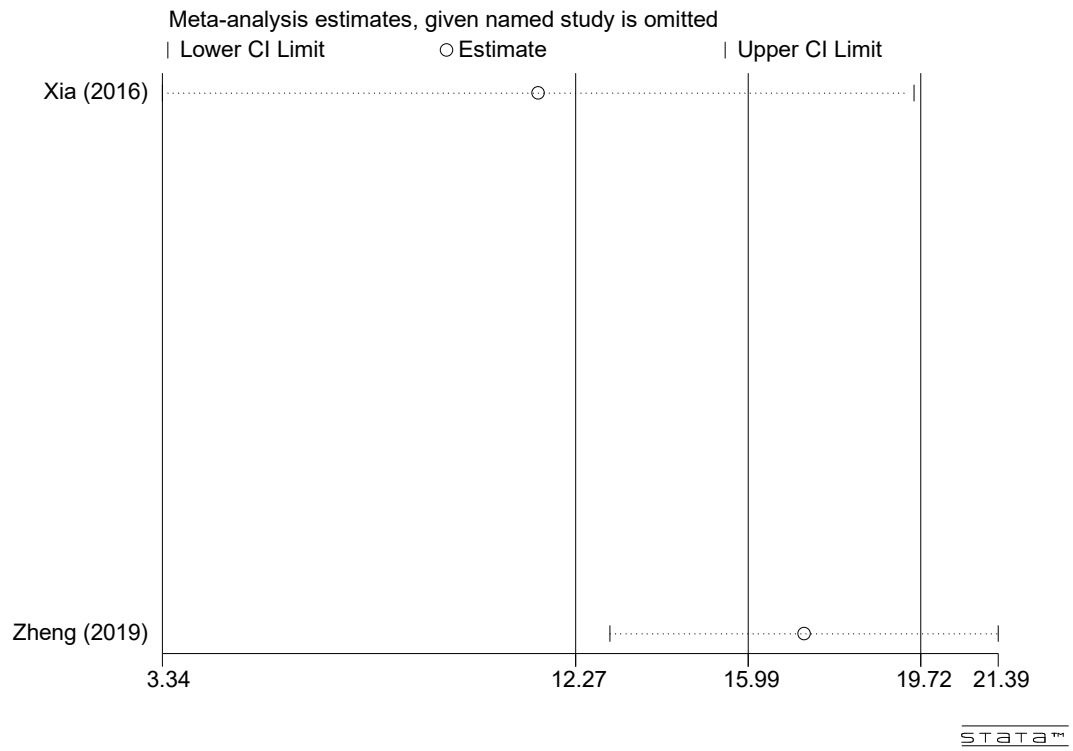

Supplement: Supplementary Materials — Table S1: search strategy. Table S2: PRISMA-P 2020 Checklist. Figure S3: the sensitivity analysis of WST of acupuncture alone. Figure S4: the sensitivity analysis of SSA of acupuncture alone. Figure S5: the sensitivity analysis of WST of acupuncture combined with rehabilitation. Figure S6: the sensitivity analysis of VFSS of acupuncture combined with rehabilitation. Figure S7: the sensitivity analysis of SSA of acupuncture combined with rehabilitation. Figure S8: the sensitivity analysis of swallowing scores of Fujishima Ichiro of acupuncture combined with rehabilitation. Figure S9: the sensitivity analysis of the rates of aspiration of acupuncture combined with rehabilitation. Figure S10: the sensitivity analysis of the rates of aspiration pneumonia of acupuncture combined with rehabilitation. Figure S11: the sensitivity analysis of DOSS of acupuncture combined with rehabilitation. Figure S12: the sensitivity analysis of BI of acupuncture combined with rehabilitation. Figure S13: the sensitivity analysis of SWAL-QOL of acupuncture combined with rehabilitation. Figure S14: the sensitivity analysis of duration of empty swallowing (submental muscle group) of acupuncture combined with rehabilitation. Figure S15: the sensitivity analysis of duration of empty swallowing (infrahyoid muscles) of acupuncture combined with rehabilitation. Figure S16: the sensitivity analysis of duration of 5 mL water swallowing (submental muscle group) of acupuncture combined with rehabilitation. Figure S17: the sensitivity analysis of duration of 5 mL water swallowing (infrahyoid muscles) of acupuncture combined with rehabilitation. [file 8803507.f1.zip › Fig.S12 The sensitivity analysis of BI of acupuncture combine with Rehabilitation (1).pdf]

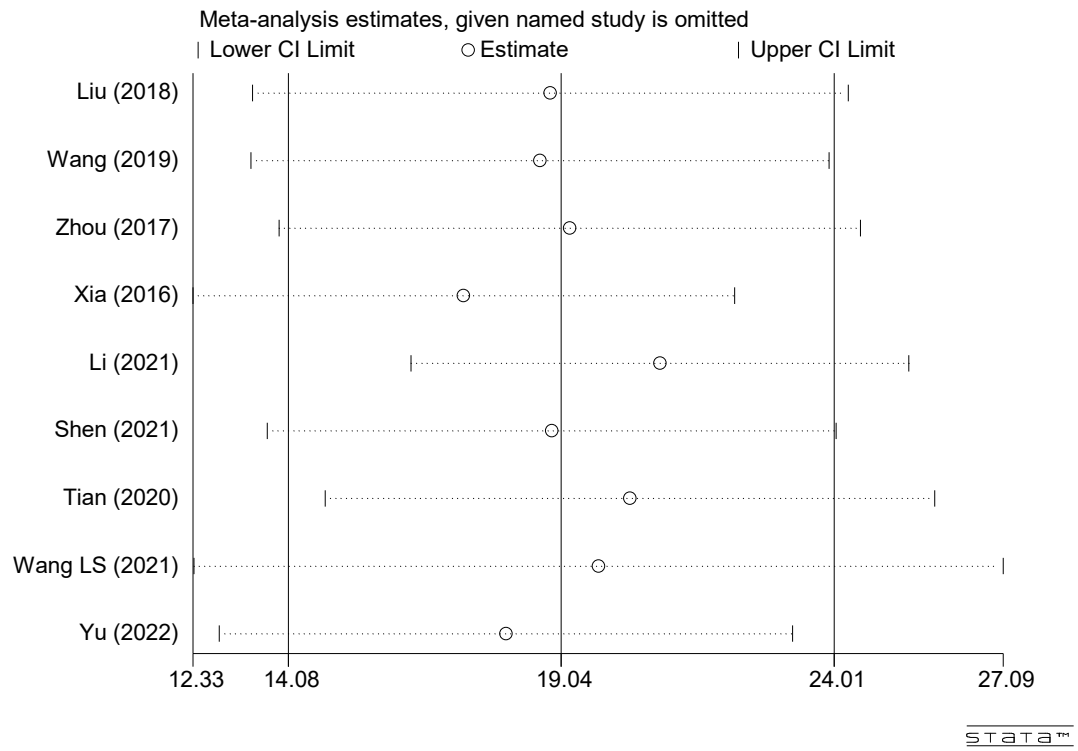

Supplement: Supplementary Materials — Table S1: search strategy. Table S2: PRISMA-P 2020 Checklist. Figure S3: the sensitivity analysis of WST of acupuncture alone. Figure S4: the sensitivity analysis of SSA of acupuncture alone. Figure S5: the sensitivity analysis of WST of acupuncture combined with rehabilitation. Figure S6: the sensitivity analysis of VFSS of acupuncture combined with rehabilitation. Figure S7: the sensitivity analysis of SSA of acupuncture combined with rehabilitation. Figure S8: the sensitivity analysis of swallowing scores of Fujishima Ichiro of acupuncture combined with rehabilitation. Figure S9: the sensitivity analysis of the rates of aspiration of acupuncture combined with rehabilitation. Figure S10: the sensitivity analysis of the rates of aspiration pneumonia of acupuncture combined with rehabilitation. Figure S11: the sensitivity analysis of DOSS of acupuncture combined with rehabilitation. Figure S12: the sensitivity analysis of BI of acupuncture combined with rehabilitation. Figure S13: the sensitivity analysis of SWAL-QOL of acupuncture combined with rehabilitation. Figure S14: the sensitivity analysis of duration of empty swallowing (submental muscle group) of acupuncture combined with rehabilitation. Figure S15: the sensitivity analysis of duration of empty swallowing (infrahyoid muscles) of acupuncture combined with rehabilitation. Figure S16: the sensitivity analysis of duration of 5 mL water swallowing (submental muscle group) of acupuncture combined with rehabilitation. Figure S17: the sensitivity analysis of duration of 5 mL water swallowing (infrahyoid muscles) of acupuncture combined with rehabilitation. [file 8803507.f1.zip › Fig.S13 The sensitivity analysis of SWAL-QOL of acupuncture combine with Rehabilitation.pdf]

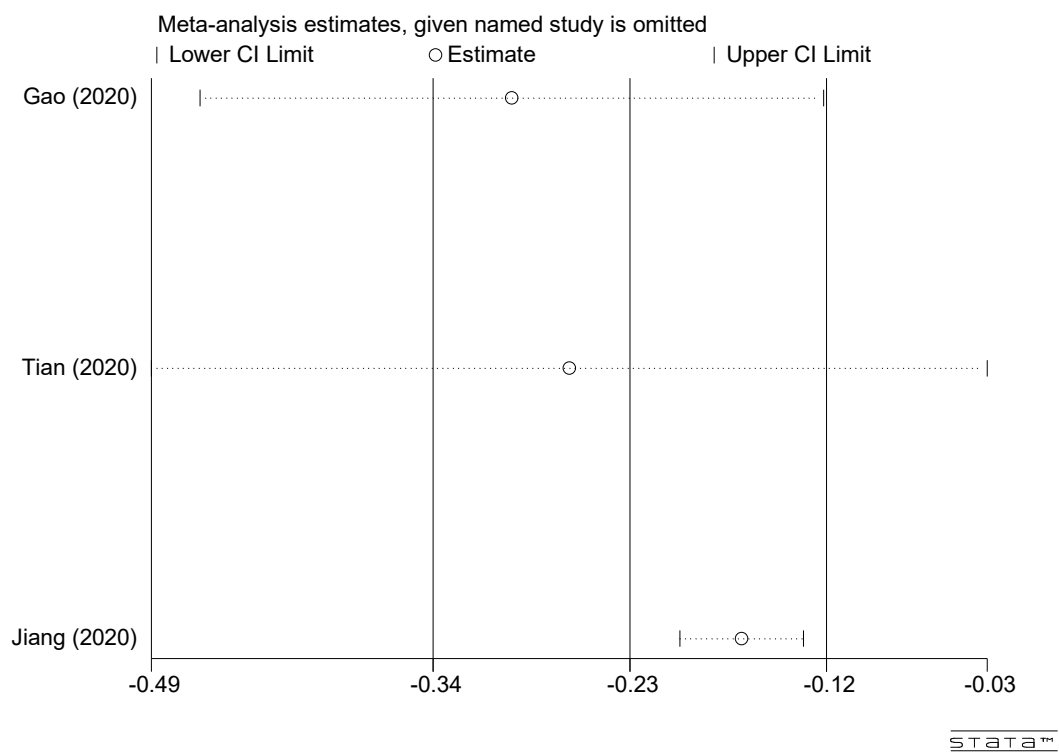

Supplement: Supplementary Materials — Table S1: search strategy. Table S2: PRISMA-P 2020 Checklist. Figure S3: the sensitivity analysis of WST of acupuncture alone. Figure S4: the sensitivity analysis of SSA of acupuncture alone. Figure S5: the sensitivity analysis of WST of acupuncture combined with rehabilitation. Figure S6: the sensitivity analysis of VFSS of acupuncture combined with rehabilitation. Figure S7: the sensitivity analysis of SSA of acupuncture combined with rehabilitation. Figure S8: the sensitivity analysis of swallowing scores of Fujishima Ichiro of acupuncture combined with rehabilitation. Figure S9: the sensitivity analysis of the rates of aspiration of acupuncture combined with rehabilitation. Figure S10: the sensitivity analysis of the rates of aspiration pneumonia of acupuncture combined with rehabilitation. Figure S11: the sensitivity analysis of DOSS of acupuncture combined with rehabilitation. Figure S12: the sensitivity analysis of BI of acupuncture combined with rehabilitation. Figure S13: the sensitivity analysis of SWAL-QOL of acupuncture combined with rehabilitation. Figure S14: the sensitivity analysis of duration of empty swallowing (submental muscle group) of acupuncture combined with rehabilitation. Figure S15: the sensitivity analysis of duration of empty swallowing (infrahyoid muscles) of acupuncture combined with rehabilitation. Figure S16: the sensitivity analysis of duration of 5 mL water swallowing (submental muscle group) of acupuncture combined with rehabilitation. Figure S17: the sensitivity analysis of duration of 5 mL water swallowing (infrahyoid muscles) of acupuncture combined with rehabilitation. [file 8803507.f1.zip › Fig.S14 The sensitivity analysis of Duration of empty swallowing(Submental muscle group) of acupuncture combine with Rehabilitation.pdf]

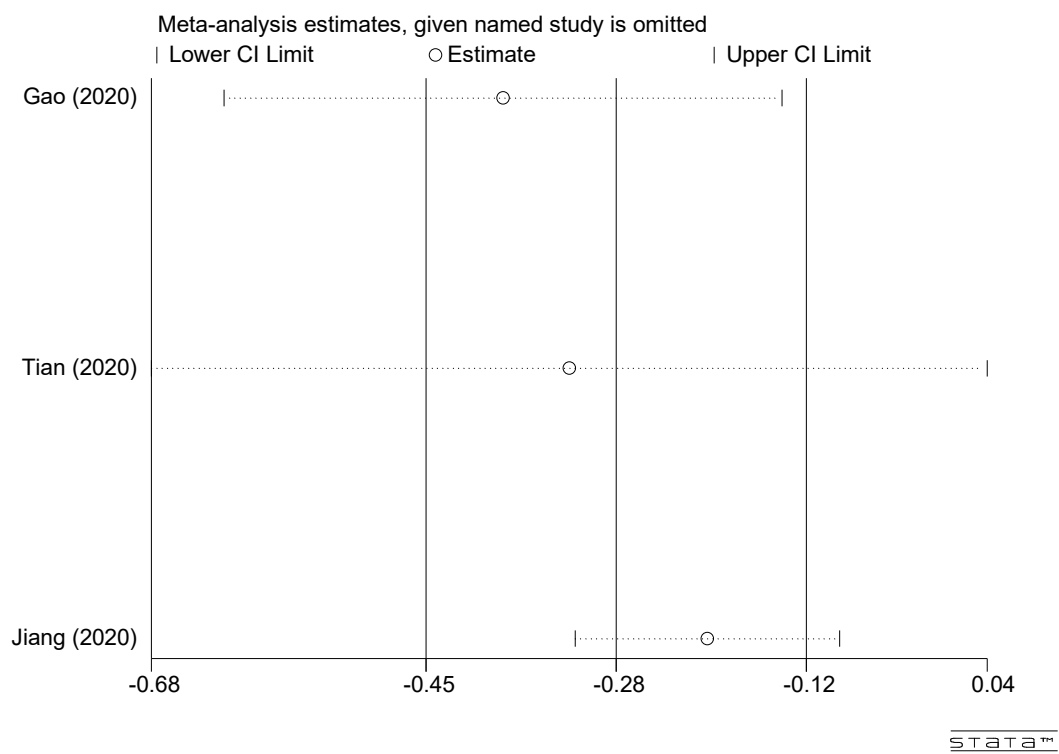

Supplement: Supplementary Materials — Table S1: search strategy. Table S2: PRISMA-P 2020 Checklist. Figure S3: the sensitivity analysis of WST of acupuncture alone. Figure S4: the sensitivity analysis of SSA of acupuncture alone. Figure S5: the sensitivity analysis of WST of acupuncture combined with rehabilitation. Figure S6: the sensitivity analysis of VFSS of acupuncture combined with rehabilitation. Figure S7: the sensitivity analysis of SSA of acupuncture combined with rehabilitation. Figure S8: the sensitivity analysis of swallowing scores of Fujishima Ichiro of acupuncture combined with rehabilitation. Figure S9: the sensitivity analysis of the rates of aspiration of acupuncture combined with rehabilitation. Figure S10: the sensitivity analysis of the rates of aspiration pneumonia of acupuncture combined with rehabilitation. Figure S11: the sensitivity analysis of DOSS of acupuncture combined with rehabilitation. Figure S12: the sensitivity analysis of BI of acupuncture combined with rehabilitation. Figure S13: the sensitivity analysis of SWAL-QOL of acupuncture combined with rehabilitation. Figure S14: the sensitivity analysis of duration of empty swallowing (submental muscle group) of acupuncture combined with rehabilitation. Figure S15: the sensitivity analysis of duration of empty swallowing (infrahyoid muscles) of acupuncture combined with rehabilitation. Figure S16: the sensitivity analysis of duration of 5 mL water swallowing (submental muscle group) of acupuncture combined with rehabilitation. Figure S17: the sensitivity analysis of duration of 5 mL water swallowing (infrahyoid muscles) of acupuncture combined with rehabilitation. [file 8803507.f1.zip › Fig.S15 The sensitivity analysis of Duration of empty swallowing(Infrahyoid muscles) of acupuncture combine with Rehabilitation.pdf]

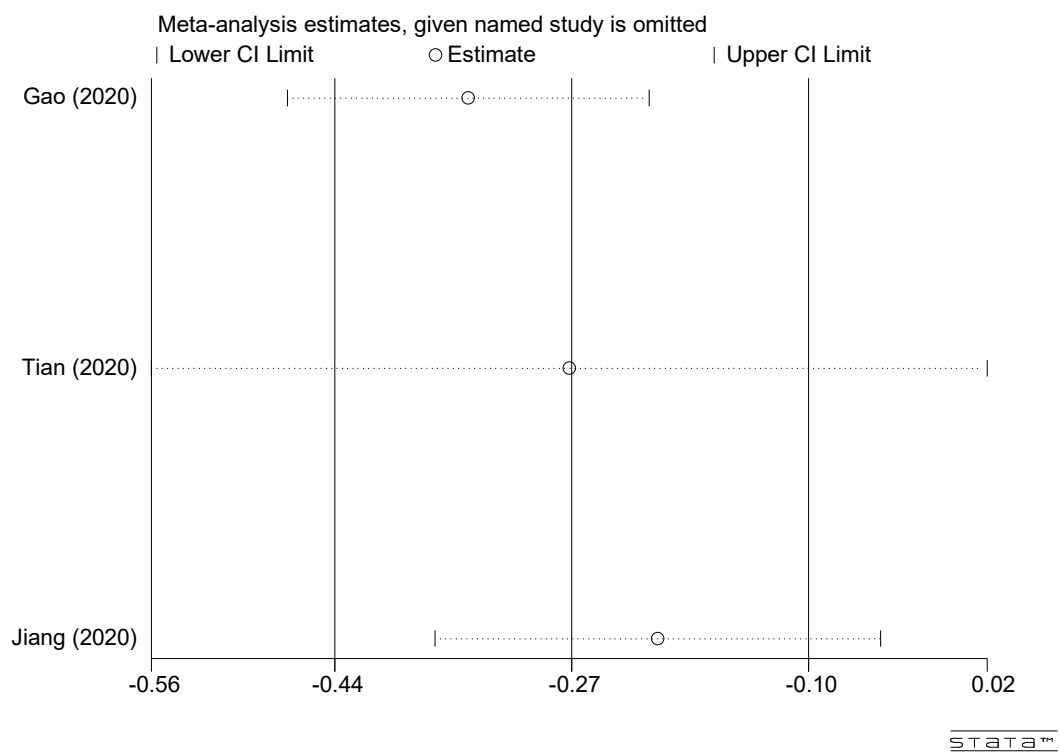

Supplement: Supplementary Materials — Table S1: search strategy. Table S2: PRISMA-P 2020 Checklist. Figure S3: the sensitivity analysis of WST of acupuncture alone. Figure S4: the sensitivity analysis of SSA of acupuncture alone. Figure S5: the sensitivity analysis of WST of acupuncture combined with rehabilitation. Figure S6: the sensitivity analysis of VFSS of acupuncture combined with rehabilitation. Figure S7: the sensitivity analysis of SSA of acupuncture combined with rehabilitation. Figure S8: the sensitivity analysis of swallowing scores of Fujishima Ichiro of acupuncture combined with rehabilitation. Figure S9: the sensitivity analysis of the rates of aspiration of acupuncture combined with rehabilitation. Figure S10: the sensitivity analysis of the rates of aspiration pneumonia of acupuncture combined with rehabilitation. Figure S11: the sensitivity analysis of DOSS of acupuncture combined with rehabilitation. Figure S12: the sensitivity analysis of BI of acupuncture combined with rehabilitation. Figure S13: the sensitivity analysis of SWAL-QOL of acupuncture combined with rehabilitation. Figure S14: the sensitivity analysis of duration of empty swallowing (submental muscle group) of acupuncture combined with rehabilitation. Figure S15: the sensitivity analysis of duration of empty swallowing (infrahyoid muscles) of acupuncture combined with rehabilitation. Figure S16: the sensitivity analysis of duration of 5 mL water swallowing (submental muscle group) of acupuncture combined with rehabilitation. Figure S17: the sensitivity analysis of duration of 5 mL water swallowing (infrahyoid muscles) of acupuncture combined with rehabilitation. [file 8803507.f1.zip › Fig.S16 The sensitivity analysis of Duration of 5 mL water swallowing(Submental muscle group) of acupuncture combine with Rehabilitation.pdf]

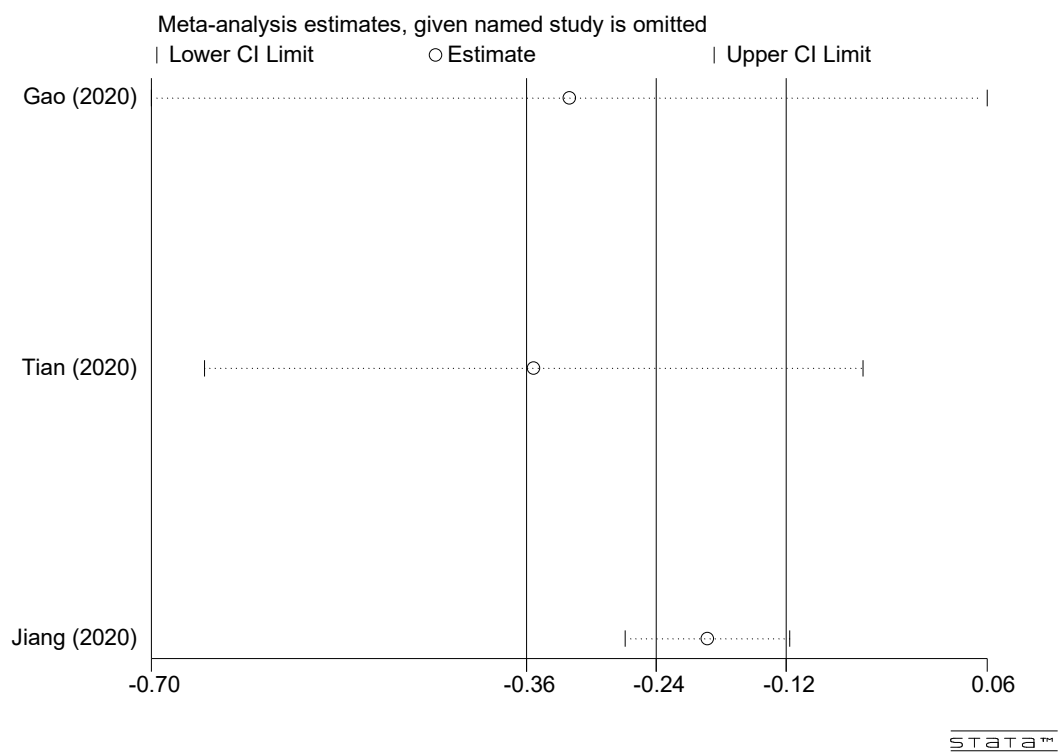

Supplement: Supplementary Materials — Table S1: search strategy. Table S2: PRISMA-P 2020 Checklist. Figure S3: the sensitivity analysis of WST of acupuncture alone. Figure S4: the sensitivity analysis of SSA of acupuncture alone. Figure S5: the sensitivity analysis of WST of acupuncture combined with rehabilitation. Figure S6: the sensitivity analysis of VFSS of acupuncture combined with rehabilitation. Figure S7: the sensitivity analysis of SSA of acupuncture combined with rehabilitation. Figure S8: the sensitivity analysis of swallowing scores of Fujishima Ichiro of acupuncture combined with rehabilitation. Figure S9: the sensitivity analysis of the rates of aspiration of acupuncture combined with rehabilitation. Figure S10: the sensitivity analysis of the rates of aspiration pneumonia of acupuncture combined with rehabilitation. Figure S11: the sensitivity analysis of DOSS of acupuncture combined with rehabilitation. Figure S12: the sensitivity analysis of BI of acupuncture combined with rehabilitation. Figure S13: the sensitivity analysis of SWAL-QOL of acupuncture combined with rehabilitation. Figure S14: the sensitivity analysis of duration of empty swallowing (submental muscle group) of acupuncture combined with rehabilitation. Figure S15: the sensitivity analysis of duration of empty swallowing (infrahyoid muscles) of acupuncture combined with rehabilitation. Figure S16: the sensitivity analysis of duration of 5 mL water swallowing (submental muscle group) of acupuncture combined with rehabilitation. Figure S17: the sensitivity analysis of duration of 5 mL water swallowing (infrahyoid muscles) of acupuncture combined with rehabilitation. [file 8803507.f1.zip › Fig.S17 The sensitivity analysis of Duration of 5 mL water swallowing(Infrahyoid muscles) of acupuncture combine with Rehabilitation.pdf]

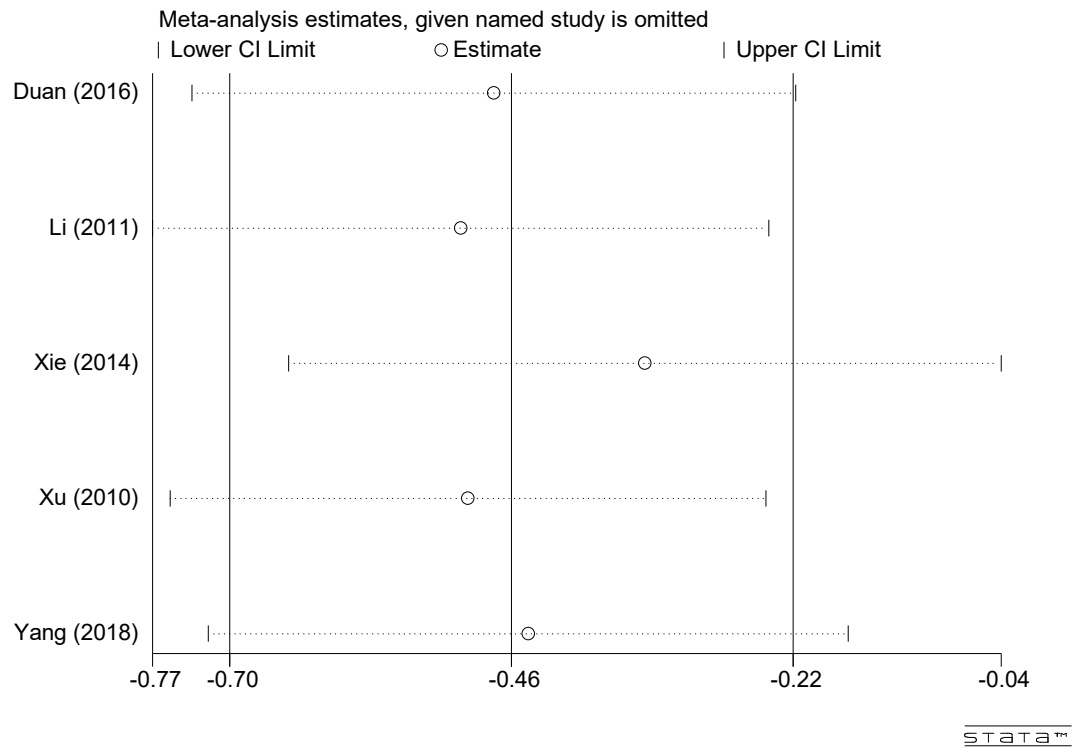

Supplement: Supplementary Materials — Table S1: search strategy. Table S2: PRISMA-P 2020 Checklist. Figure S3: the sensitivity analysis of WST of acupuncture alone. Figure S4: the sensitivity analysis of SSA of acupuncture alone. Figure S5: the sensitivity analysis of WST of acupuncture combined with rehabilitation. Figure S6: the sensitivity analysis of VFSS of acupuncture combined with rehabilitation. Figure S7: the sensitivity analysis of SSA of acupuncture combined with rehabilitation. Figure S8: the sensitivity analysis of swallowing scores of Fujishima Ichiro of acupuncture combined with rehabilitation. Figure S9: the sensitivity analysis of the rates of aspiration of acupuncture combined with rehabilitation. Figure S10: the sensitivity analysis of the rates of aspiration pneumonia of acupuncture combined with rehabilitation. Figure S11: the sensitivity analysis of DOSS of acupuncture combined with rehabilitation. Figure S12: the sensitivity analysis of BI of acupuncture combined with rehabilitation. Figure S13: the sensitivity analysis of SWAL-QOL of acupuncture combined with rehabilitation. Figure S14: the sensitivity analysis of duration of empty swallowing (submental muscle group) of acupuncture combined with rehabilitation. Figure S15: the sensitivity analysis of duration of empty swallowing (infrahyoid muscles) of acupuncture combined with rehabilitation. Figure S16: the sensitivity analysis of duration of 5 mL water swallowing (submental muscle group) of acupuncture combined with rehabilitation. Figure S17: the sensitivity analysis of duration of 5 mL water swallowing (infrahyoid muscles) of acupuncture combined with rehabilitation. [file 8803507.f1.zip › Fig.S3 The sensitivity analysis of WST of acupuncture alone (1).pdf]

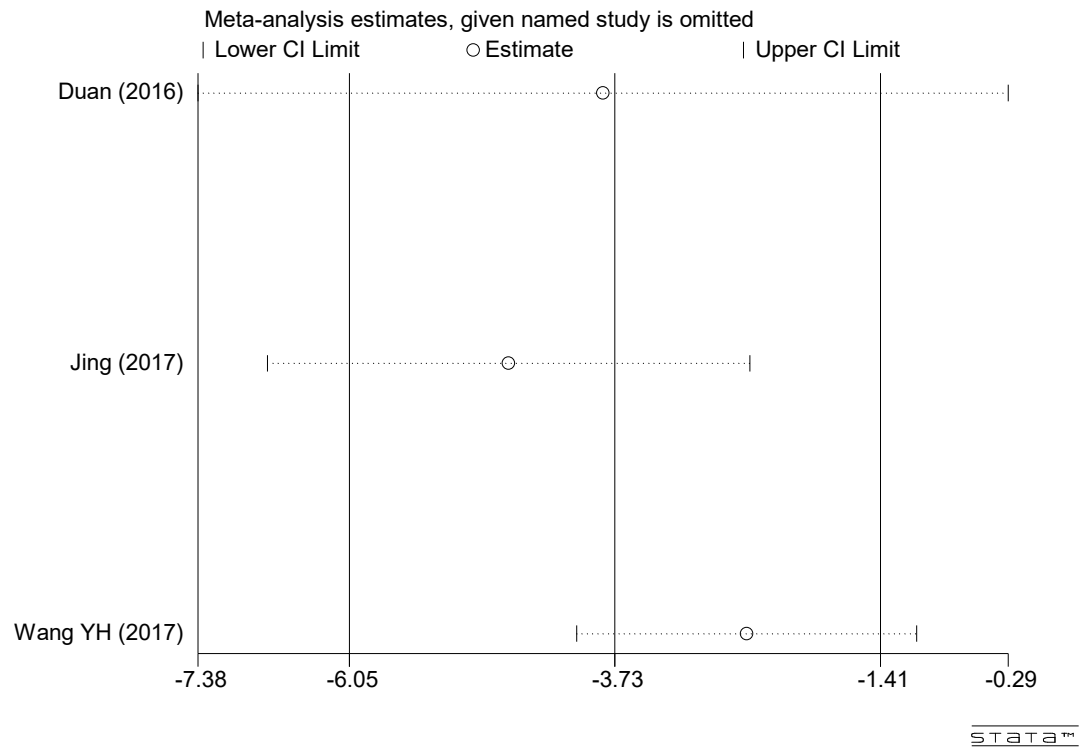

Supplement: Supplementary Materials — Table S1: search strategy. Table S2: PRISMA-P 2020 Checklist. Figure S3: the sensitivity analysis of WST of acupuncture alone. Figure S4: the sensitivity analysis of SSA of acupuncture alone. Figure S5: the sensitivity analysis of WST of acupuncture combined with rehabilitation. Figure S6: the sensitivity analysis of VFSS of acupuncture combined with rehabilitation. Figure S7: the sensitivity analysis of SSA of acupuncture combined with rehabilitation. Figure S8: the sensitivity analysis of swallowing scores of Fujishima Ichiro of acupuncture combined with rehabilitation. Figure S9: the sensitivity analysis of the rates of aspiration of acupuncture combined with rehabilitation. Figure S10: the sensitivity analysis of the rates of aspiration pneumonia of acupuncture combined with rehabilitation. Figure S11: the sensitivity analysis of DOSS of acupuncture combined with rehabilitation. Figure S12: the sensitivity analysis of BI of acupuncture combined with rehabilitation. Figure S13: the sensitivity analysis of SWAL-QOL of acupuncture combined with rehabilitation. Figure S14: the sensitivity analysis of duration of empty swallowing (submental muscle group) of acupuncture combined with rehabilitation. Figure S15: the sensitivity analysis of duration of empty swallowing (infrahyoid muscles) of acupuncture combined with rehabilitation. Figure S16: the sensitivity analysis of duration of 5 mL water swallowing (submental muscle group) of acupuncture combined with rehabilitation. Figure S17: the sensitivity analysis of duration of 5 mL water swallowing (infrahyoid muscles) of acupuncture combined with rehabilitation. [file 8803507.f1.zip › Fig.S4 The sensitivity analysis of SSA of acupuncture alone (1).pdf]

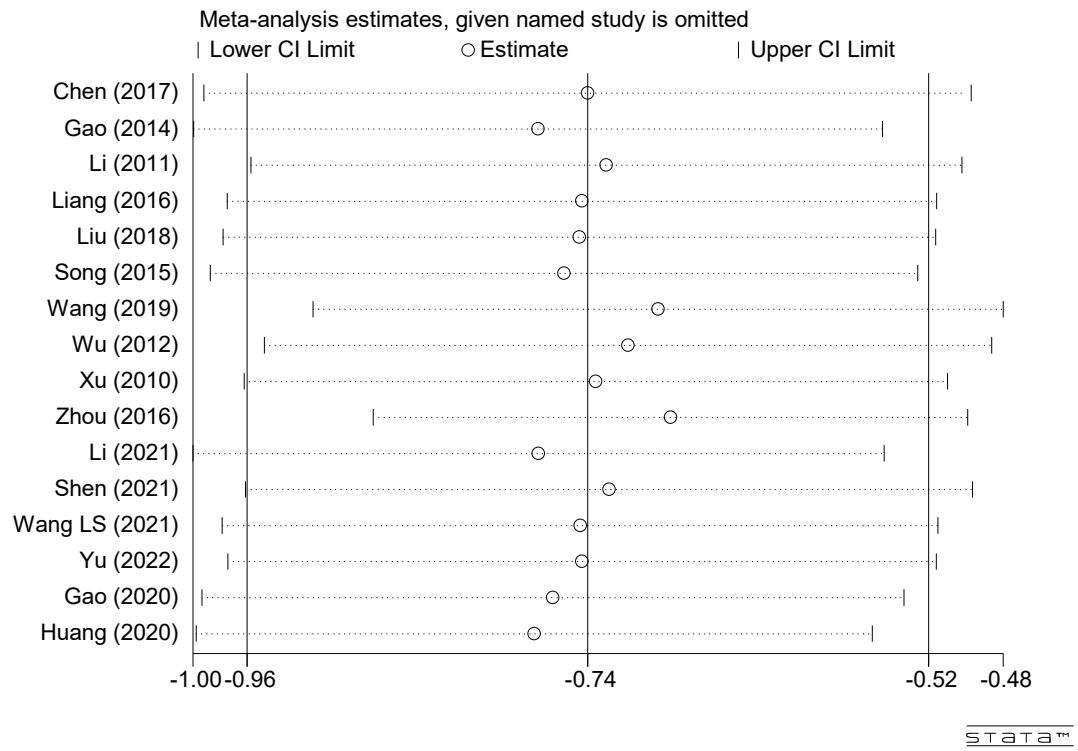

Supplement: Supplementary Materials — Table S1: search strategy. Table S2: PRISMA-P 2020 Checklist. Figure S3: the sensitivity analysis of WST of acupuncture alone. Figure S4: the sensitivity analysis of SSA of acupuncture alone. Figure S5: the sensitivity analysis of WST of acupuncture combined with rehabilitation. Figure S6: the sensitivity analysis of VFSS of acupuncture combined with rehabilitation. Figure S7: the sensitivity analysis of SSA of acupuncture combined with rehabilitation. Figure S8: the sensitivity analysis of swallowing scores of Fujishima Ichiro of acupuncture combined with rehabilitation. Figure S9: the sensitivity analysis of the rates of aspiration of acupuncture combined with rehabilitation. Figure S10: the sensitivity analysis of the rates of aspiration pneumonia of acupuncture combined with rehabilitation. Figure S11: the sensitivity analysis of DOSS of acupuncture combined with rehabilitation. Figure S12: the sensitivity analysis of BI of acupuncture combined with rehabilitation. Figure S13: the sensitivity analysis of SWAL-QOL of acupuncture combined with rehabilitation. Figure S14: the sensitivity analysis of duration of empty swallowing (submental muscle group) of acupuncture combined with rehabilitation. Figure S15: the sensitivity analysis of duration of empty swallowing (infrahyoid muscles) of acupuncture combined with rehabilitation. Figure S16: the sensitivity analysis of duration of 5 mL water swallowing (submental muscle group) of acupuncture combined with rehabilitation. Figure S17: the sensitivity analysis of duration of 5 mL water swallowing (infrahyoid muscles) of acupuncture combined with rehabilitation. [file 8803507.f1.zip › Fig.S5 The sensitivity analysis of WST of acupuncture combine with Rehabilitation.pdf]

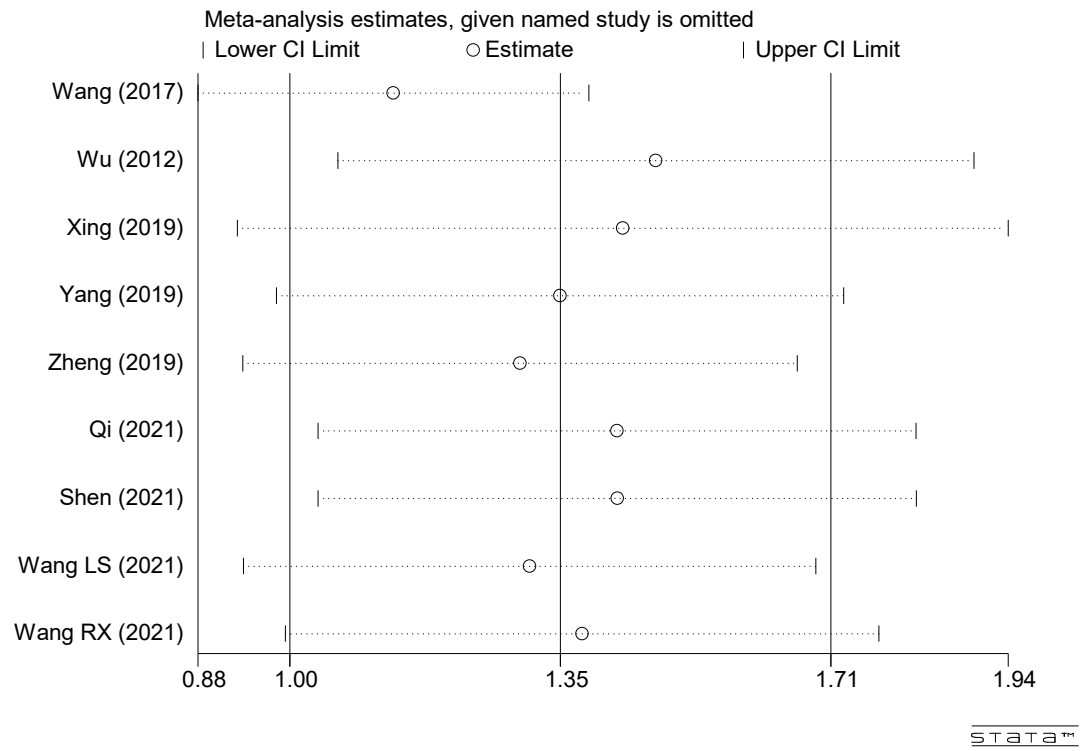

Supplement: Supplementary Materials — Table S1: search strategy. Table S2: PRISMA-P 2020 Checklist. Figure S3: the sensitivity analysis of WST of acupuncture alone. Figure S4: the sensitivity analysis of SSA of acupuncture alone. Figure S5: the sensitivity analysis of WST of acupuncture combined with rehabilitation. Figure S6: the sensitivity analysis of VFSS of acupuncture combined with rehabilitation. Figure S7: the sensitivity analysis of SSA of acupuncture combined with rehabilitation. Figure S8: the sensitivity analysis of swallowing scores of Fujishima Ichiro of acupuncture combined with rehabilitation. Figure S9: the sensitivity analysis of the rates of aspiration of acupuncture combined with rehabilitation. Figure S10: the sensitivity analysis of the rates of aspiration pneumonia of acupuncture combined with rehabilitation. Figure S11: the sensitivity analysis of DOSS of acupuncture combined with rehabilitation. Figure S12: the sensitivity analysis of BI of acupuncture combined with rehabilitation. Figure S13: the sensitivity analysis of SWAL-QOL of acupuncture combined with rehabilitation. Figure S14: the sensitivity analysis of duration of empty swallowing (submental muscle group) of acupuncture combined with rehabilitation. Figure S15: the sensitivity analysis of duration of empty swallowing (infrahyoid muscles) of acupuncture combined with rehabilitation. Figure S16: the sensitivity analysis of duration of 5 mL water swallowing (submental muscle group) of acupuncture combined with rehabilitation. Figure S17: the sensitivity analysis of duration of 5 mL water swallowing (infrahyoid muscles) of acupuncture combined with rehabilitation. [file 8803507.f1.zip › Fig.S6 The sensitivity analysis of VFSS of acupuncture combine with Rehabilitation (1).pdf]

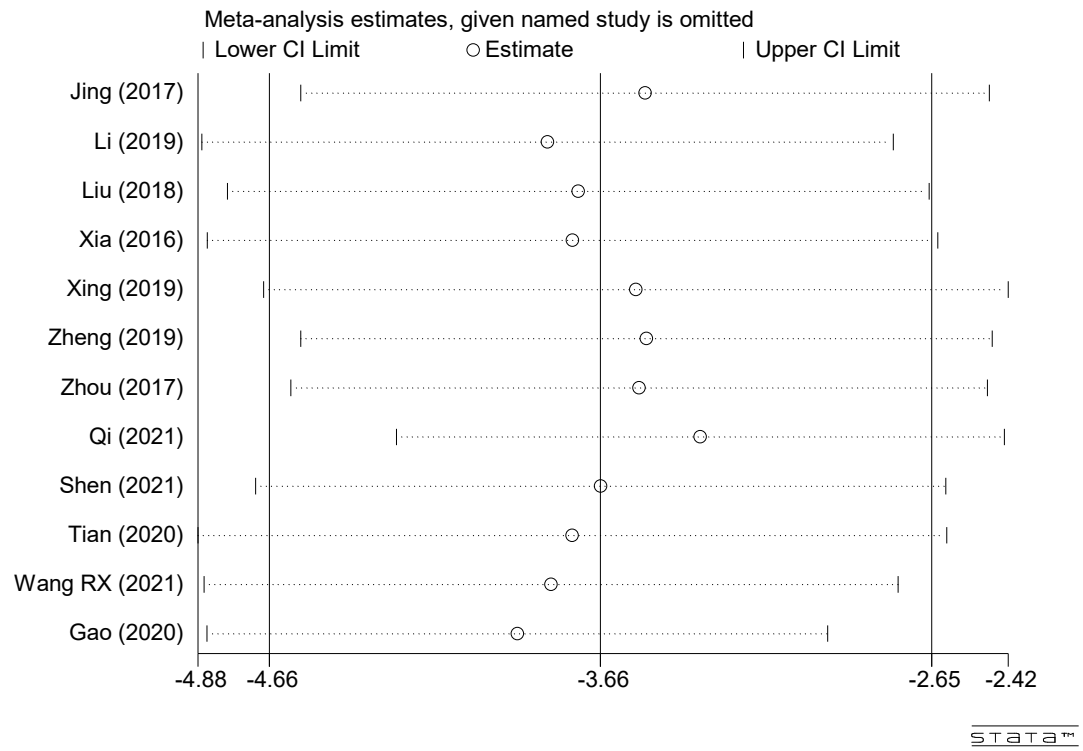

Supplement: Supplementary Materials — Table S1: search strategy. Table S2: PRISMA-P 2020 Checklist. Figure S3: the sensitivity analysis of WST of acupuncture alone. Figure S4: the sensitivity analysis of SSA of acupuncture alone. Figure S5: the sensitivity analysis of WST of acupuncture combined with rehabilitation. Figure S6: the sensitivity analysis of VFSS of acupuncture combined with rehabilitation. Figure S7: the sensitivity analysis of SSA of acupuncture combined with rehabilitation. Figure S8: the sensitivity analysis of swallowing scores of Fujishima Ichiro of acupuncture combined with rehabilitation. Figure S9: the sensitivity analysis of the rates of aspiration of acupuncture combined with rehabilitation. Figure S10: the sensitivity analysis of the rates of aspiration pneumonia of acupuncture combined with rehabilitation. Figure S11: the sensitivity analysis of DOSS of acupuncture combined with rehabilitation. Figure S12: the sensitivity analysis of BI of acupuncture combined with rehabilitation. Figure S13: the sensitivity analysis of SWAL-QOL of acupuncture combined with rehabilitation. Figure S14: the sensitivity analysis of duration of empty swallowing (submental muscle group) of acupuncture combined with rehabilitation. Figure S15: the sensitivity analysis of duration of empty swallowing (infrahyoid muscles) of acupuncture combined with rehabilitation. Figure S16: the sensitivity analysis of duration of 5 mL water swallowing (submental muscle group) of acupuncture combined with rehabilitation. Figure S17: the sensitivity analysis of duration of 5 mL water swallowing (infrahyoid muscles) of acupuncture combined with rehabilitation. [file 8803507.f1.zip › Fig.S7 The sensitivity analysis of SSA of acupuncture combine with Rehabilitation (1).pdf]

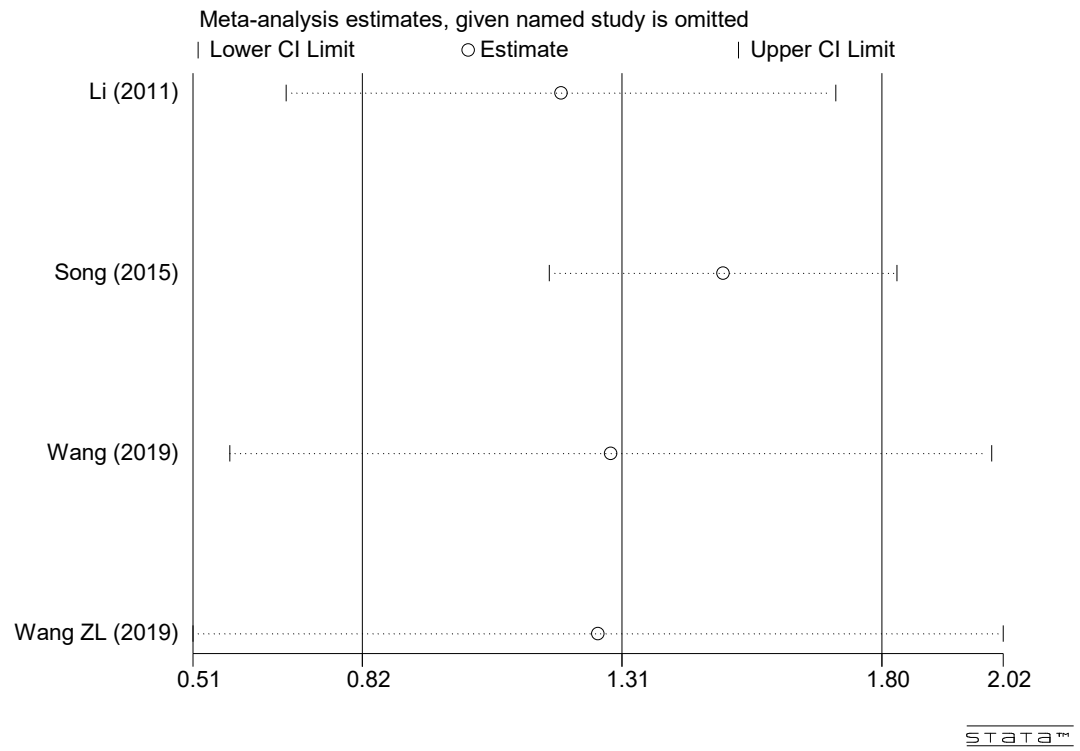

Supplement: Supplementary Materials — Table S1: search strategy. Table S2: PRISMA-P 2020 Checklist. Figure S3: the sensitivity analysis of WST of acupuncture alone. Figure S4: the sensitivity analysis of SSA of acupuncture alone. Figure S5: the sensitivity analysis of WST of acupuncture combined with rehabilitation. Figure S6: the sensitivity analysis of VFSS of acupuncture combined with rehabilitation. Figure S7: the sensitivity analysis of SSA of acupuncture combined with rehabilitation. Figure S8: the sensitivity analysis of swallowing scores of Fujishima Ichiro of acupuncture combined with rehabilitation. Figure S9: the sensitivity analysis of the rates of aspiration of acupuncture combined with rehabilitation. Figure S10: the sensitivity analysis of the rates of aspiration pneumonia of acupuncture combined with rehabilitation. Figure S11: the sensitivity analysis of DOSS of acupuncture combined with rehabilitation. Figure S12: the sensitivity analysis of BI of acupuncture combined with rehabilitation. Figure S13: the sensitivity analysis of SWAL-QOL of acupuncture combined with rehabilitation. Figure S14: the sensitivity analysis of duration of empty swallowing (submental muscle group) of acupuncture combined with rehabilitation. Figure S15: the sensitivity analysis of duration of empty swallowing (infrahyoid muscles) of acupuncture combined with rehabilitation. Figure S16: the sensitivity analysis of duration of 5 mL water swallowing (submental muscle group) of acupuncture combined with rehabilitation. Figure S17: the sensitivity analysis of duration of 5 mL water swallowing (infrahyoid muscles) of acupuncture combined with rehabilitation. [file 8803507.f1.zip › Fig.S8 The sensitivity analysis of swallowing scores of Fujishima Ichiro of acupuncture combine with Rehabilitation (1).pdf]

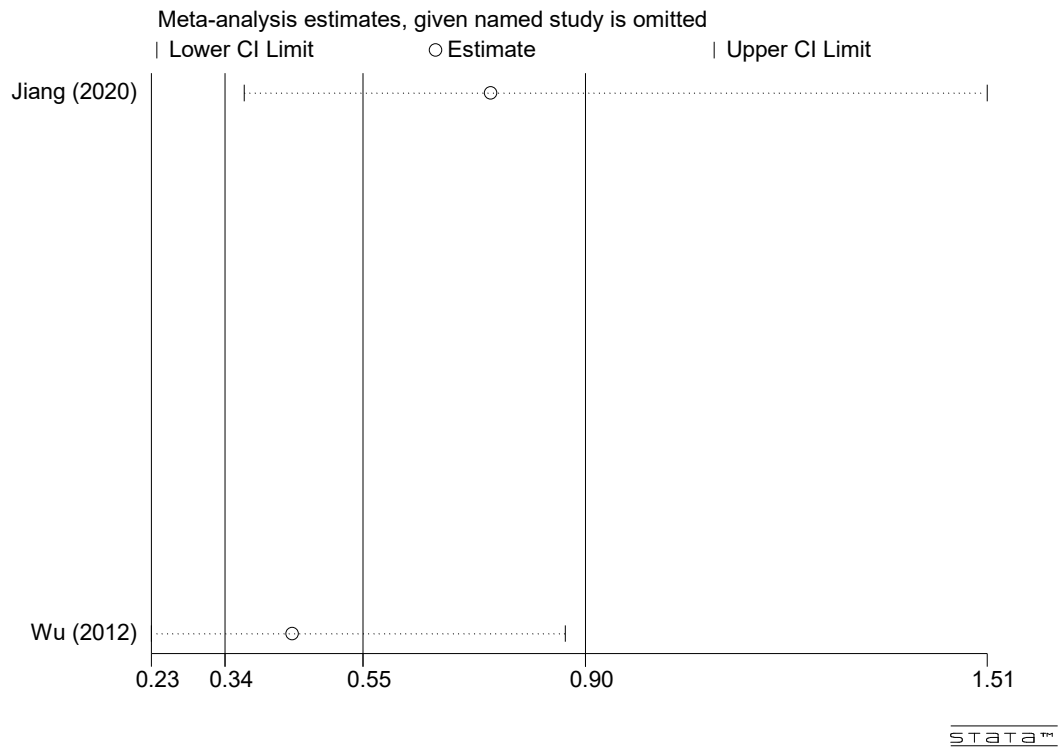

Supplement: Supplementary Materials — Table S1: search strategy. Table S2: PRISMA-P 2020 Checklist. Figure S3: the sensitivity analysis of WST of acupuncture alone. Figure S4: the sensitivity analysis of SSA of acupuncture alone. Figure S5: the sensitivity analysis of WST of acupuncture combined with rehabilitation. Figure S6: the sensitivity analysis of VFSS of acupuncture combined with rehabilitation. Figure S7: the sensitivity analysis of SSA of acupuncture combined with rehabilitation. Figure S8: the sensitivity analysis of swallowing scores of Fujishima Ichiro of acupuncture combined with rehabilitation. Figure S9: the sensitivity analysis of the rates of aspiration of acupuncture combined with rehabilitation. Figure S10: the sensitivity analysis of the rates of aspiration pneumonia of acupuncture combined with rehabilitation. Figure S11: the sensitivity analysis of DOSS of acupuncture combined with rehabilitation. Figure S12: the sensitivity analysis of BI of acupuncture combined with rehabilitation. Figure S13: the sensitivity analysis of SWAL-QOL of acupuncture combined with rehabilitation. Figure S14: the sensitivity analysis of duration of empty swallowing (submental muscle group) of acupuncture combined with rehabilitation. Figure S15: the sensitivity analysis of duration of empty swallowing (infrahyoid muscles) of acupuncture combined with rehabilitation. Figure S16: the sensitivity analysis of duration of 5 mL water swallowing (submental muscle group) of acupuncture combined with rehabilitation. Figure S17: the sensitivity analysis of duration of 5 mL water swallowing (infrahyoid muscles) of acupuncture combined with rehabilitation. [file 8803507.f1.zip › Fig.S9 The sensitivity analysis of The rates ofaspiration of acupuncture combine with Rehabilitation (1).pdf]
